# Reversible enzyme-catalysed NAD^+^/NADH electrochemistry†

**DOI:** 10.1039/d5sc00570a

**Published:** 2025-03-04

**Authors:** Peter D. Giang, Dimitri Niks, Sheron Hakopian, Russ Hille, Paul V. Bernhardt

**Affiliations:** a School of Chemistry and Molecular Biosciences, University of Queensland Brisbane 4072 Australia p.bernhardt@uq.edu.au; b Department of Biochemistry, University of California Riverside USA

## Abstract

Formate dehydrogenase (FdsDABG) from *Cupriavidus necator* is a Mo-containing enzyme capable of catalysing both formate oxidation to CO_2_ and the reverse CO_2_ reduction to formate by utilising NAD^+^ or NADH, respectively. This enzyme is part of the NADH dehydrogenase superfamily. Its subcomplex, FdsBG, lacking the formate oxidizing/CO_2_-reducing Mo-cofactor, but harbouring an FMN as well as [2Fe–2S] and [4Fe–4S] clusters, reversibly interconverts the NAD^+^/NADH redox pair. UV-vis spectroelectrochemistry across the range 6 < pH < 8 determined the redox potentials of these three cofactors. Cyclic voltammetry was used to explore mechanistic and kinetic properties of each oxidation- and reduction-half reaction. Through mediated enzyme electrochemistry experiments, the Michaelis constant for NADH oxidation (*K*_M,NADH_ = 1.7 × 10^2^ μM) was determined using methylene blue as a redox mediator. For the reverse NAD^+^ reduction reaction using methyl viologen as electron donor a similar analysis yielded the value of *K*_M,NAD^+^_ = 1.2 mM. All experimental voltammetry data were reproduced by electrochemical simulations furnishing a set of self-consistent rate constants for the catalytic FdsBG system for both NAD^+^ reduction and NADH oxidation. This comprises the first electrochemical kinetic analysis of its kind for a reversible NADH dehydrogenase enzyme and provides new insight to the function of the FdsDABG formate dehydrogenase holoenzyme.

## Abstract 2

The formate dehydrogenase subcomplex FdsBG from *Cupriavidus necator* has been electrochemically activated to reversibly interconvert NAD^+^/NADH and provide unique insight to its kinetics and reactivity.

## Graphical abstract

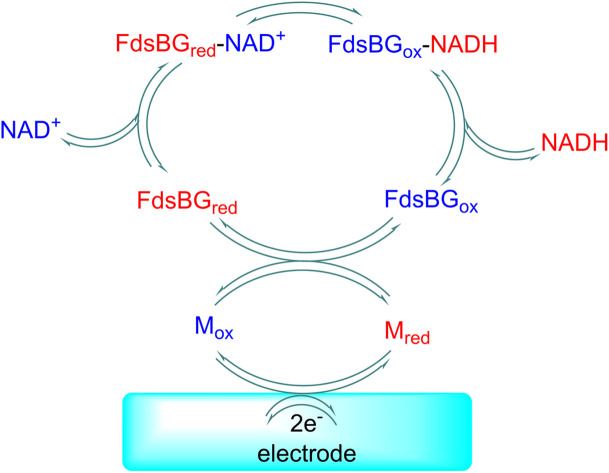

## Introduction

Reduced nicotinamide adenine dinucleotide (NADH) and its phosphate analogue NADPH are ubiquitous and essential reductants involved in numerous biochemical pathways principally as hydride donors. Given the widespread use of NAD(P)H as a reductant of many enzymes and its commercial cost, there is interest in its regeneration,^1^ but the selective non-biological reduction of NAD(P)^+^ to NAD(P)H (1,4-dihydro isomer) is difficult. Non-biologically active byproducts (1,2- and 1,6-dihydro isomers and NADH dimers) are a particular issue.^2,3^ In contrast to synthetic methods,^4^ many oxidoreductase enzymes are capable of regenerating NAD(P)H from NAD(P)^+^ with high specificity and efficiency, including glucose dehydrogenase,^5^ hydrogenase,^6^ alcohol dehydrogenase,^7,8^ phosphite dehydrogenase^9,10^ and formate dehydrogenase.^11–13^ Despite multiple enzyme candidates for this reaction, typical biochemical problems exist for some of these enzymes that limit scale-up. Utilisation of these enzymes for NAD(P)H regeneration to support other enzyme systems introduces complexity, with multiple active enzymes and co-substrates present.^14^ Further drawbacks include the difficulty in selectively separating out NAD(P)H from a complex enzymatic mixture which may still include structurally similar NAD(P)^+^.^15^ Furthermore, the aforementioned enzyme candidates have displayed limited pH-, temperature-, and air-stability.^16–18^

**Fig. 1:**
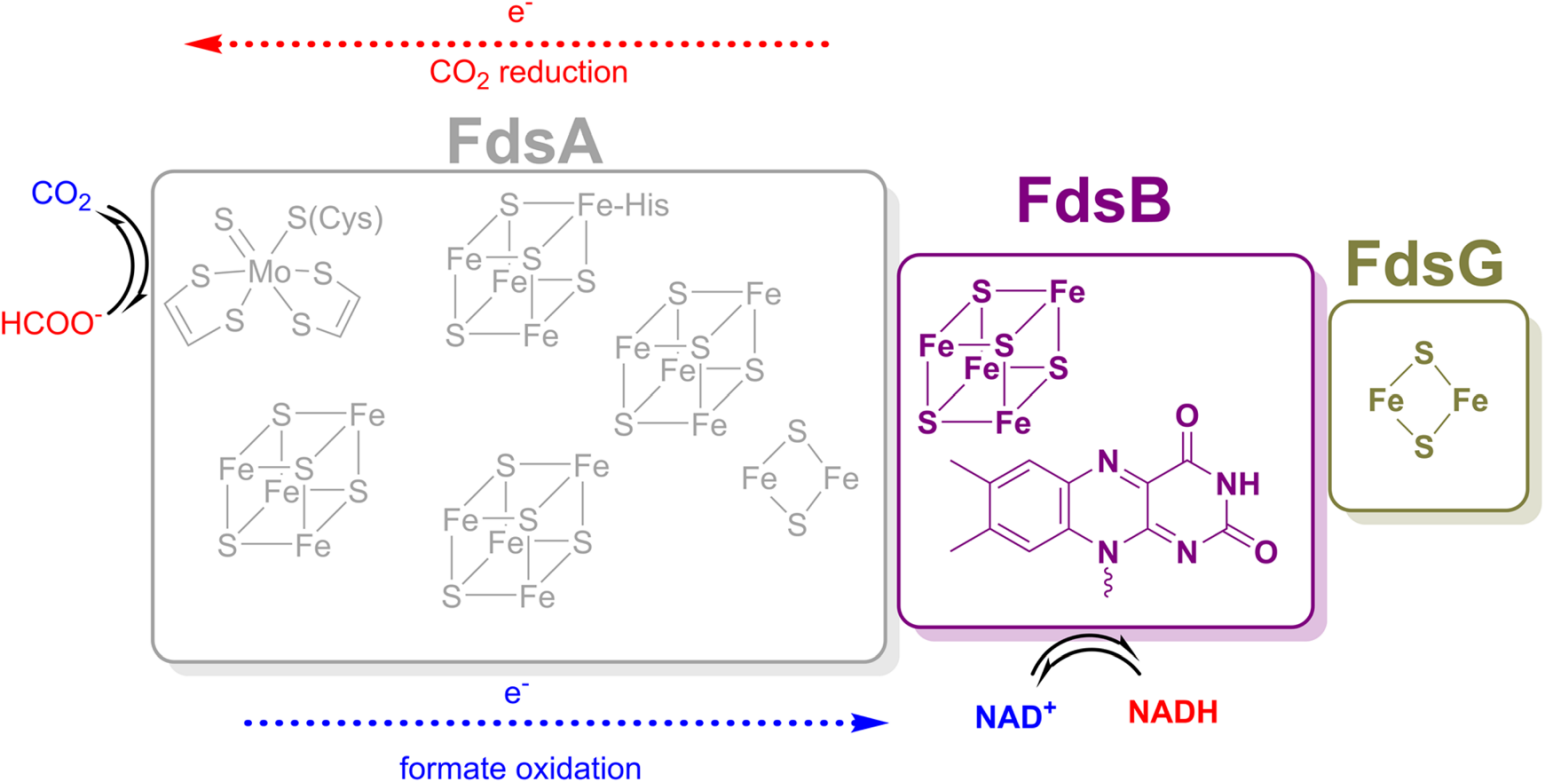
Cartoon representation of the FdsA, FdsB, and FdsG subunits and their respective cofactors of FdsDABG from *C. necator*. The FdsD subunit is not shown as it does not contain any cofactors.

One enzyme of particular interest is the Mo-containing formate dehydrogenase (FdsDABG) from the Gram-negative soil bacterium *Cupriavidus necator* (*C. necator*), which is air-stable, can reversibly catalyse both formate oxidation to CO_2_ and CO_2_ reduction to formate, and is also a member of the NADH dehydrogenase family.^19^ Given its stability and catalytic ability, FdsDABG has been explored in biotechnological applications.^20–22^ Cryo-EM studies of FdsDABG from *Rhodobacter capsulatus* (*R. capsulatus*), which shares high sequence homology with FdsDABG from *C. necator*, revealed the spatial disposition of these cofactors within the heterotetrameric protomer which is illustrated in cartoon form in Fig. 1.^23^ The FdsA subunit (105 kDa) contains the molybdenum centre (the site of formate oxidation and CO_2_ reduction), one [2Fe–2S] cluster and four [4Fe–4S] clusters. The FdsB subunit (55 kDa) contains one [4Fe–4S] cluster and a flavin mononucleotide (FMN), which is the NADH/NAD^+^ binding site. The FdsG subunit (19 kDa) contains a single [2Fe–2S] cluster.^24^ The small (7 kDa) FdsD subunit (not shown in Fig. 1) has no redox-active cofactors but is thought to be involved in Mo cofactor maturation and insertion.^25^

The combination of structural complexity and relative oxygen tolerance of FdsDABG has prompted investigations into its mechanism and function.^26^ Recent redox characterisation of the cofactors in *C. necator* FdsDABG was achieved through a combination of EPR-monitored redox potentiometry and optical spectroelectrochemistry on both the holoenzyme FdsDABG and its subcomplex FdsBG.^27^ The FdsBG subcomplex has also been crystallographically characterised.^24^ A partial redox characterisation of the homologous FdsDABG from *R. capsulatus* has subsequently been reported.^28^

The focus of this study is electrochemical activation of the FdsBG subcomplex as a reversible NAD^+^/NADH oxidoreductase. Isolated FdsBG exhibits NADH:O_2_ oxidoreductase (diaphorase) activity,^24^ although within the FdsDABG holoenzyme the native function of the (reduced) FMN cofactor is to reduce NAD^+^ rather than oxidise NADH. The system thus presents an ideal example of a bidirectional NADH/NAD^+^ redox system. To date, investigations into NAD^+^ reduction and NADH oxidation have only been performed through steady-state and stopped flow single-turnover kinetic experiments.^19,22,24,26^ The electrochemical approach taken here removes the need for chemical reductants (dithionite) which may obscure important spectroscopic information. We have also explored the use of both single- and two-electron redox mediators and have undertaken a full kinetic analysis using electrochemical simulations. This study represents a new approach to expensive nicotinamide recycling, which remains an area of considerable biotechnological importance.^29,30^ The mechanistic information emerging from this analysis reveals previously inaccessible kinetic data that not only explains the high NADH/NAD^+^ recycling activity of the FdsBG subcomplex but also the FdsDABG holoenzyme.

## Experimental

### Protein purification

The FdsBG subcomplex was expressed, purified, and characterised according to a published procedure^24^ and was stored frozen (−80 °C) in phosphate buffer (50 mM, pH 7.5) at a concentration of 178 μM.

### Reagents

The organic mediators phenazinium (PMS, methosulfate salt), methylene blue (MB^+^, chloride salt), methyl viologen (MV^2+^, chloride salt) and Safranin T (chloride salt) were all obtained commercially. Transition metal mediators for spectroelectrochemistry included [Fe(*trans*-diammac)](ClO_4_)_3_,^31^ [Co(AMMEN_4_S_2_sar)]Cl_3_,^32^ [Co(AMMEN_5_Ssar)]Cl_3_,^33^ [Co(sep)]Cl_3_,^34^ [Co(AMMEsar)]Cl_3_,^35^ [Co(ClMeClAbsar)]Cl_3_,^35^ and [Co(*cis*-diammac)](ClO_4_)_3_.^36^ The structures of all mediators are given in the ESI (Fig. S1).† All other reagents were obtained commercially at analytical grade and were used as purchased. All solutions were prepared using ultrapure water (18.2 MΩ cm).

### UV-vis spectroelectrochemistry

Optical (UV-vis) spectroelectrochemical experiments were performed in a quartz spectroelectrochemical cell (1.7 mm path length) with a gold “honeycomb” working electrode (Pine Instruments), a gold auxiliary electrode and a Ag/AgCl reference electrode calibrated with quinhydrone at pH 7 (*E*_m,7_ +0.285 V *vs.* NHE). The cell contained an approximately 600 μL solution comprising FdsBG (approximately 100 μM), and redox mediators. These included [Fe(*trans*-diammac)]^3+^, [Co(AMMEN_4_S_2_sar)]^3+^, [Co(AMMEN_5_Ssar)]^3+^, [Co(sep)]^3+^, [Co(AMMEsar)]^3+^, [Co(ClMeClAbsar)]^3+^, and [Co(*cis*-diammac)]^3+^ (each 80 μM). The M^III/II^ (M = Fe, Co) redox couples of these complexes are reversible and their potentials span the oxidation–reduction potential (ORP) range 0 to −0.55 V *vs.* NHE (Fig. S1†). They also exhibit low molar absorption coefficients in the visible region (*ε* < 500 M^−1^ cm^−1^) resulting in negligible spectral interference at the micromolar concentrations used.^37,38^ UV-vis spectra were acquired with an Agilent 8453 diode array UV-vis spectrophotometer, with the cell cooled to 15 °C by a Huber Ministat 230 refrigerated water-circulating bath. Applied potentials were controlled by a Gamry Interface 1010 potentiostat. UV-vis spectra were recorded at 12.5 mV intervals when electrochemical equilibrium was established and no further spectral changes were occurring. Reversibility was established upon returning from either more negative to more positive (or from more positive to more negative) potential with minimal spectral hysteresis. For single electron redox reactions, the relationship between spectral absorbance and applied potential (*E*) is given by a combination of the Nernst equation and Beer–Lambert law as seen in eqn (1). In this equation, *A*_ox_ and *A*_red_ are the absorbances at that wavelength of the oxidised and reduced forms of the chromophore, and *E*_m_ is the midpoint potential (V).1
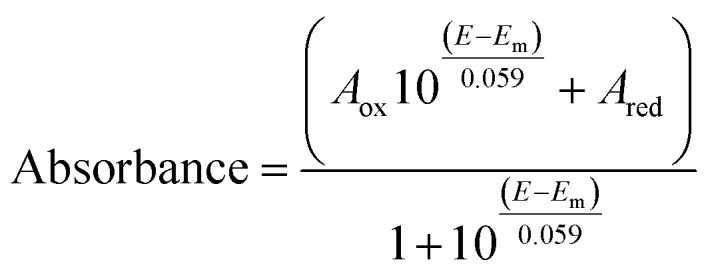


For two electron redox reactions, eqn (2) applies where *E*_1_ and *E*_2_ are the potentials of the first and second reductions. *A*_ox_, *A*_int_, and *A*_red_ are the absorbances at a given wavelength of the three redox states.2
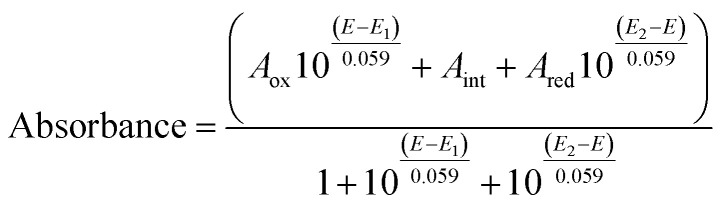


The potential-dependent spectra were modelled by global analysis with ReactLab Redox^39^ that fits the potential dependent absorbance data (at all wavelengths) to eqn (1) and (2). These data are collected in Table 1. All experimental data are given in the ESI (Fig. S2)† with spectral analyses at pH 6 (Fig. S3†) and pH 8 (Fig. S4†).

**Table 1:** Redox potentials (*V vs.* NHE) of the [4Fe–4S], FMN and [2Fe–2S] cofactors present in FdsBG at different pH values as determined through optical spectroelectrochemistry. In this study, pH 6, 7 and 8 are new data. Data for pH 7.5 are from previous work^27^

	pH 6	pH 7	pH 7.5 (ref. 27)	pH 8
[4Fe–4S]^2+/+^	—	−0.45	−0.49	−0.48
FMN/FMNH˙	−0.24	−0.25	−0.30	−0.32
FMNH˙/FMNH^−^	−0.31	−0.34	−0.38	−0.37
[2Fe–2S]^2+/+^ (deflavo form^a^)	−0.12 (−0.15)	−0.15 (−0.19)	−0.13 (−0.21)	−0.13 (−0.20)

a Tentative assignment of the more negative [2Fe–2S]^2+/+^ redox potential to deflavo-FdsBG.

### Electrochemical measurements

Cyclic voltammetry (CV) experiments were conducted at 25 °C with a BASi EC Epsilon EClipse potentiostat using a three-electrode system comprising a glassy carbon disk working electrode, platinum wire counter electrode, and Ag/AgCl reference electrode. The glassy carbon electrode was polished mechanically in a slurry of alumina (0.05 μm) in ultrapure water on a microfibre polishing pad. The electrode was then sonicated in ultrapure water for 15 minutes to give a mirror-like finish.

The electroactive surface of the glassy carbon electrode was calculated from the cyclic voltammogram of 1 mM ferrocene methanol in 0.1 M KCl solution at multiple scan rates using the Randles–Sevcik equation (eqn (3)).^40^ The diffusion coefficient (*D*) of ferrocene methanol is 6.7 × 10^−6^ cm^2^ s^−1^,^41^*i*_p_ is the CV current maximum, *n* is the number of electrons (here *n* = 1), *C* is the concentration of analyte (mol cm^−3^), and *υ* is the sweep rate (V s^−1^). The surface area was determined to be *A* = 0.056 cm^2^.

3 *i*_p_ = 2.69 × 10^5^*D*^1/2^*n*^3/2^*ACυ*^1/2^

The diffusion coefficients of the methyl viologen dication/radical monocation (MV^2+/+^˙) and the methylene blue monocation/neutral leuco form (MB^+^/MBH) were also determined using eqn (3) and the above value of *A* in a similar fashion. Linear regression of *i*_p_*versus υ*^1/2^ gave values of *D*(MV^2+/+^˙) = 3.8 × 10^−6^ cm^2^ s^−1^ and *D*(MB^+^/MBH) = 2.1 × 10^−6^ cm^2^ s^−1^, which are consistent with published work.^42,43^

### Catalytic voltammetry

The electrochemical solution (2.5 mL 50 mM phosphate, pH 7.5) contained methyl viologen (60 μM) or methylene blue (20 μM) and was purged with N_2_. These concentrations were sufficiently high to obtain a good signal to noise ratio. Higher mediator concentrations gave relatively smaller changes as a function of substrate concentration in accord with theory.^44^ FdsBG (0.34 μM) was added to the solution and mixed by gentle stirring (50 rpm) under a blanket of N_2_. Small aliquots of stock solutions of NAD^+^ or NADH were added and mixed to give bulk concentrations described in the text. Cyclic voltammograms were then run on quiescent solutions at scan rates from 2 to 20 mV s^−1^. The catalytic currents (*i*_lim_) were taken as the nett current once the contribution from the mediator current (at zero substrate concentration) was subtracted (at −0.55 V for MV^2+^ or +0.15 V for MB^+^). The currents were modelled as a function of substrate (*S*) concentration with the Michaelis–Menten equation (eqn (4)).4
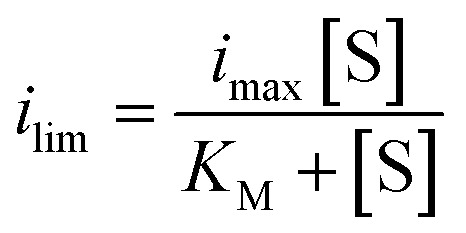
where *i*_max_, the limiting current at saturating concentration of substrate (proportional to the turnover number) and *K*_M_ is the Michaelis constant.^45^ Under a given set of conditions (pH, enzyme and mediator concentrations) *i*_max_ will be a constant.

### pH dependent measurements

Seven different buffer solutions (each 50 mM) were prepared, with pH values ranging from 6 to 9 in 0.5 pH unit increments adjusted with dilute NaOH or AcOH: citrate (pH 6), phosphate (pH 6.5, pH 7, and pH 7.5), bicine (pH 8 and pH 8.5), and CHES (2-(cyclohexylamino)ethane-1-sulfonic acid, pH 9). All pH measurements were made and rechecked *in situ* in the electrochemical cell with a Hanna 8424 pH meter and Hanna HI-1093B pH electrode (Microbulb).

For NAD^+^ reduction experiments, to each of the above buffer solutions (2.5 mL) was added methyl viologen (12.5 μL of 10 mM) to give a final concentration of 60 μM. The solution was purged with N_2_ for a few minutes, the gas inlet tube was withdrawn from the solution and a blanket of N_2_ was maintained throughout the experiment. FdsBG was added (5 μL of 178 μM) to give a final concentration of 0.34 μM (active enzyme) after gently mixing with a magnetic stirrer (50 rpm). Stirring was discontinued and a CV was measured at a scan rate of 5 mV s^−1^ which was used as a baseline measurement (zero activity). Under a blanket of N_2_, NAD^+^ (125 μL of 100 mM) was added and mixed with a magnetic stirrer for 10 min under a blanket of N_2_ to give a 5 mM solution. Stirring was discontinued and the CV was measured.

The above procedure was repeated for all buffer solutions at their pH value. The differences in cathodic current at −0.55 V *vs.* NHE with and without NAD^+^ was taken as the value of *i*_max_ and these data as a function of pH were fit to eqn (5) which is applicable for an enzyme that is deactivated by protonation of a base at acidic pH values (p*K*_a2_) or deprotonation of an acid at basic pH values (p*K*_a1_), and *i*_opt_ is the catalytic current at the optimal pH ((p*K*_a1_ + p*K*_a2_)/2).5
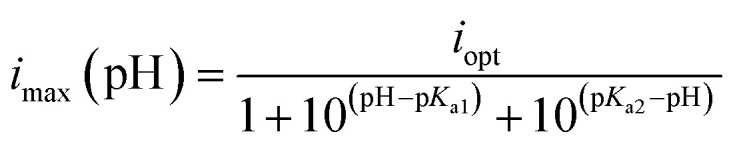


For NADH oxidation experiments, methylene blue (5 μL of 10 mM) was added to each of the above buffer solutions (2.5 mL) to give a final concentration of 20 μM. As above, the solution was purged with N_2_, the gas supply tube was withdrawn from the solution whilst maintaining a blanket of N_2_ then FdsBG was added (5 μL of 178 μM) to give a final concentration of 0.34 μM (active enzyme). A CV was measured then NADH (125 μL of 10 mM) was added with gentle stirring under a N_2_ blanket to give a final concentration of 500 μM. After 10 min stirring was discontinued and a CV was measured. This was repeated at each pH value and the current differences at +0.15 V *vs.* NHE with and without NADH were fit to eqn (5).

### Electrochemical simulation

**Scheme 1:**
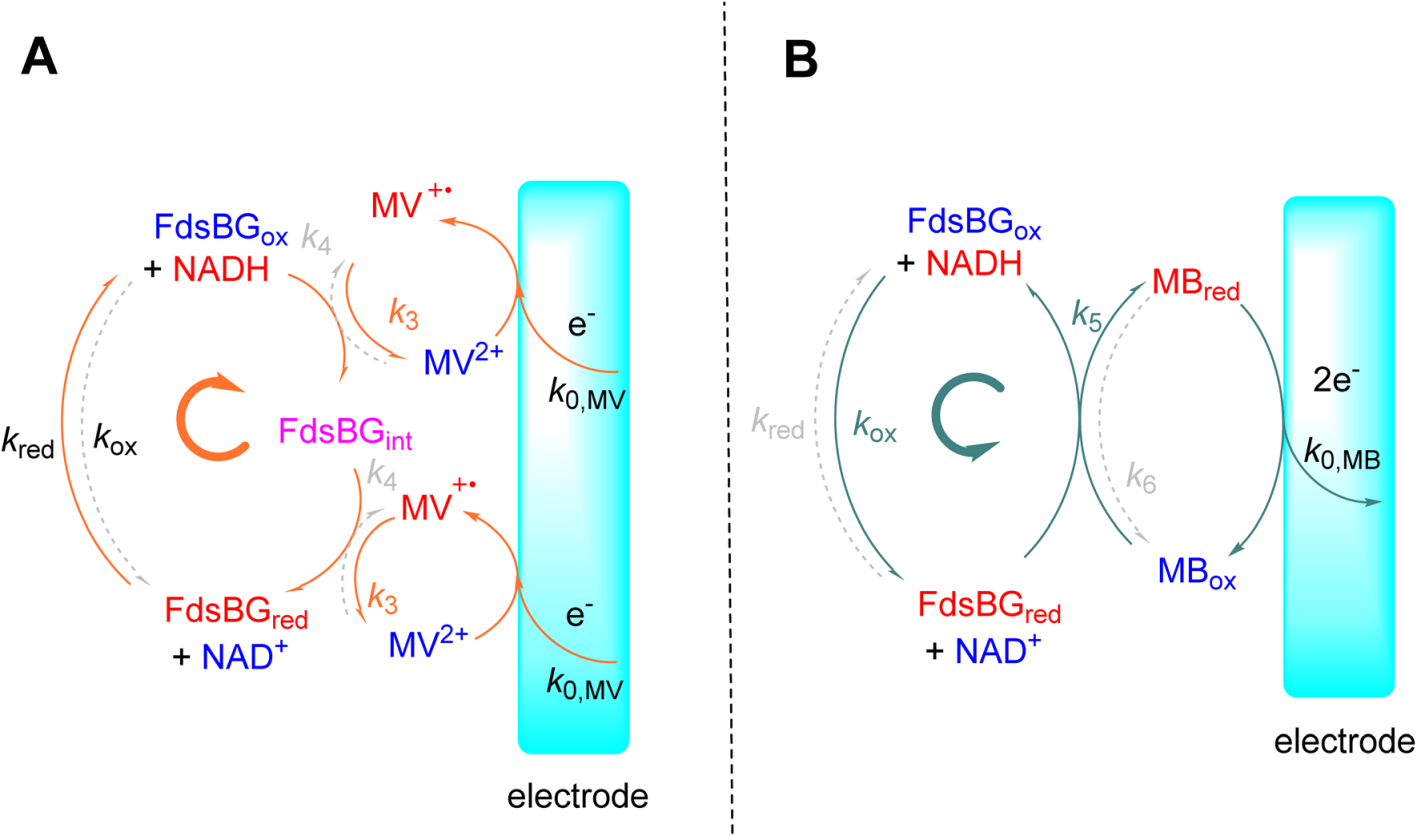
The model employed for electrochemical simulations of FdsBG-catalysed (A) NAD^+^ reduction mediated by methyl viologen (orange solid arrows showing clockwise direction of cycle) and (B) NADH oxidation mediated by methylene blue (grey solid arrows – anticlockwise direction). The grey broken arrows show the reverse (unfavored) directions of each catalytic cycle.

The Digisim program (version 3.03b) was utilised to simulate the experimental cyclic voltammetry data.^46^ The models for NAD^+^ reduction/NADH oxidation are shown in Scheme 1. Cyclic voltammograms were acquired on solutions containing known concentrations of FdsBG, NAD^+^/NADH and mediator methyl viologen or methylene blue at scan rates in the range 2 to 20 mV s^−1^. The redox potentials of all mediators were determined from control experiments without FdsBG or substrate present. The diffusion coefficients of mediators MV^2+/+^˙ (3.8 × 10^−6^ cm^2^ s^−1^) and MB^+^/MBH (2.1 × 10^−6^ cm^2^ s^−1^) were determined by CV herein as described above. A value of *D*(NAD^+^/NADH) = 4.0 × 10^−6^ cm^2^ s^−1^ was taken from the literature.^47^ The diffusion coefficient of FdsBG is unknown but its crystal structure^24^ reveals a roughly spherical shape so based on its molecular weight (*M* = 74 000 Da) a diffusion coefficient may be estimated^48^ using eqn 6.6
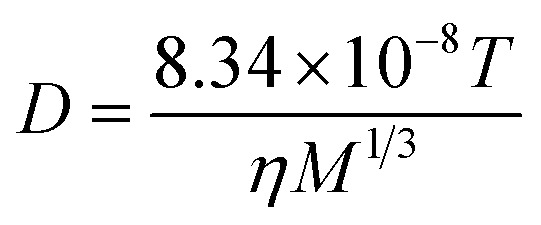
where *T* is the temperature (K) and *η* is the viscosity of the solvent (water 1.00 cP). A value of *D*(FdsBG) = 5.9 × 10^−7^ cm^2^ s^−1^ was calculated (for all redox states). The heterogeneous rate constants for MV^2+/+^˙ and MB^+^/MBH were obtained by simulation of the CV data in the absence of substrate and values of *k*_0_ = 1 × 10^−2^ cm s^−1^ were obtained. The most important kinetic parameters are given in Table 2 while all simulation parameters are assembled in the ESI (Table S1).† The same set of rate constants reproduced 16 voltammograms measured at different scan rates and substrate concentrations (Fig. 7 and 8) so the rate constants represent a consensus set of values with uncertainties estimated to be 20% as reported previously.^49^

Kinetic parameters derived from electrochemical simulation of FdsBG-catalysed NAD^+^ reduction and NADH oxidation (see Scheme 1). Values in italics uncertain due to the reactions favouring MV^+^˙ oxidation/MB^+^ reduction (*k*_3_ ≫ *k*_4_ and *k*_5_ ≫ *k*_6_). Proton transfer reactions are omitted for clarity

**Table:** 

Mediator independent		
FdsBG_red_ + NAD^+^ ⇌ FdsBG_ox_ + NADH	*k*_red_ 2.0 × 10^6^ M^−1^ s^−1^	*k*_ox_ 2.0 × 10^6^ M^−1^ s^−1^

a *k*_3_ assumed to be equal for reduction of FdsBG_ox_ and FdsBG_int_.

b *k*_4_ assumed to equal for oxidation of FdsBG_int_ and FdsBG_red_.

**Table:** 

Mediator dependent		
MV^+^˙ + FdsBG_ox_ ⇌ MV^2+^ + FdsBG_int_	*k*_3_ 3.5 × 10^7^ M^−1^ s^−1^	*k*_4_ *3.5 × 10* ^*4*^ M^−1^ s^−1^
MV^+^˙ + FdsBG_int_ ⇌ MV^2+^ + FdsBG_red_	*k*_3_ a 3.5 × 10^7^ M^−1^ s^−1^	*k*_4_ b *3.5 × 10* ^*4*^ M^−1^ s^−1^
MB^+^ + FdsBG_red_ ⇌ MBH + FdsBG_ox_	*k*_5_ 1.6 × 10^7^ M^−1^ s^−1^	*k*_6_ *1.6 × 10* ^*3*^ M^−1^ s^−1^

Given the symmetry of the NAD^+^ reduction/NADH oxidation reaction (Scheme 1A and B), the main mechanistic differences between FdsBG-catalysed NAD^+^ reduction and NADH oxidation are that NAD^+^ reduction comprises two consecutive one-electron reductions by MV^+^˙ *via* an intermediate oxidation state (FdsBG_int_) while the 2-electron, MB-mediated NADH oxidation bypasses the intermediate FdsBG oxidation state. We have assumed the rates of the consecutive single electron transfer steps (*k*_3_/*k*_4_) are equal as their driving forces are essentially the same and the site of binding will be the same regardless of whether FdsBG_ox_ or FdsBG_int_ is reacting. The two FMN redox potentials (FMN/FMNH˙ and FMNH˙/FMNH^−^) lie between those of the [2Fe–2S]^2+/+^ and [4Fe–4S]^2+/+^ couples (Table 1) and NAD^+^ reduction/NADH oxidation must occur at the fully reduced/oxidised FMN active site, respectively. The model does not consider redox reactions at the [2Fe–2S]^2+/+^ and [4Fe–4S]^2+/+^ cofactors (intermolecular of intramolecular) which are unproductive in terms of substrate turnover at the FMN cofactor.

## Results and discussion

### Structural aspects

**Fig. 2:**
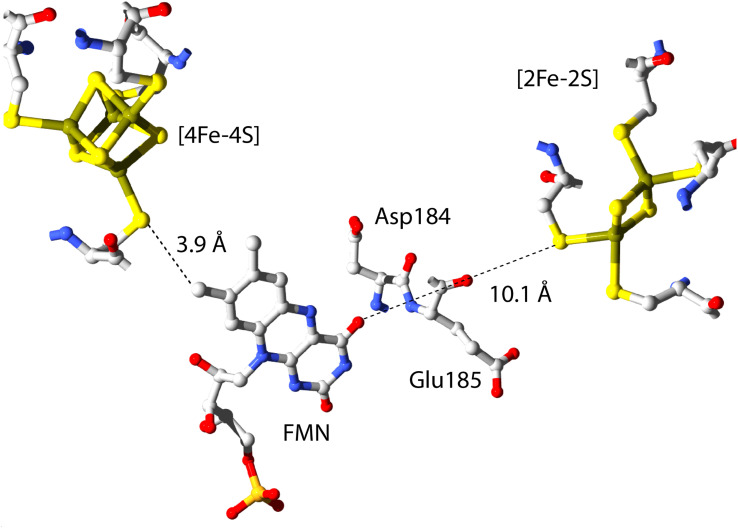
The relative positions of the three redox active cofactors in FdsBG. Coordinates were taken from the published X-ray crystal structure (PDB 6VW7).^24^

The cofactor positions within FdsBG have been established by X-ray crystallography^24^ and this is shown in Fig. 2. Within the FdsB subunit, the [4Fe–4S] and FMN cofactors are in proximity (∼3.9 Å) and trace the final two steps in electron transfer from the molybdenum centre (in the FdsA subunit of the FdsDABG holoenzyme) to NAD^+^ following formate oxidation (see Fig. 1). The FdsB [4Fe–4S] cluster lies close to the [2Fe–2S] cluster of FdsA in the holoenzyme (Fig. 1) and is ‘on-path’ for electron transfer from the molybdenum centre to FMN. The [2Fe–2S] cluster (in the FdsG subunit) is ‘off-path’ but still within electron transfer range (∼10.1 Å) of the FMN. The amino acid residues Asp184 and Glu185 (in the FdsG subunit) are situated between the FMN and [2Fe–2S] cluster. The FMN cofactor is where NAD^+^/NADH binds, and this has also been demonstrated crystallographically.^24^

### pH-dependent UV-vis spectroelectrochemistry

The three redox-active cofactors in Fig. 2 comprise a two-electron FMN/FMNH˙/FMNH^−^ couple and one-electron [4Fe–4S]^2+/+^ and [2Fe–2S]^2+/+^ couples. Each cofactor in its oxidised form influences the absorbance spectrum of FdsBG and they undergo typical UV-vis spectral changes upon reduction and oxidation. In their fully reduced forms the FMNH^−^, [4Fe–4S]^+^ and [2Fe–2S]^+^ cofactors become largely bleached throughout the UV-visible region. The redox potentials of FdsBG at pH 7.5 are known^27^ but here we have extended this investigation over the pH range 6 to 8 to support pH-dependent electrochemical studies (presented in the next section).

**Fig. 3:**
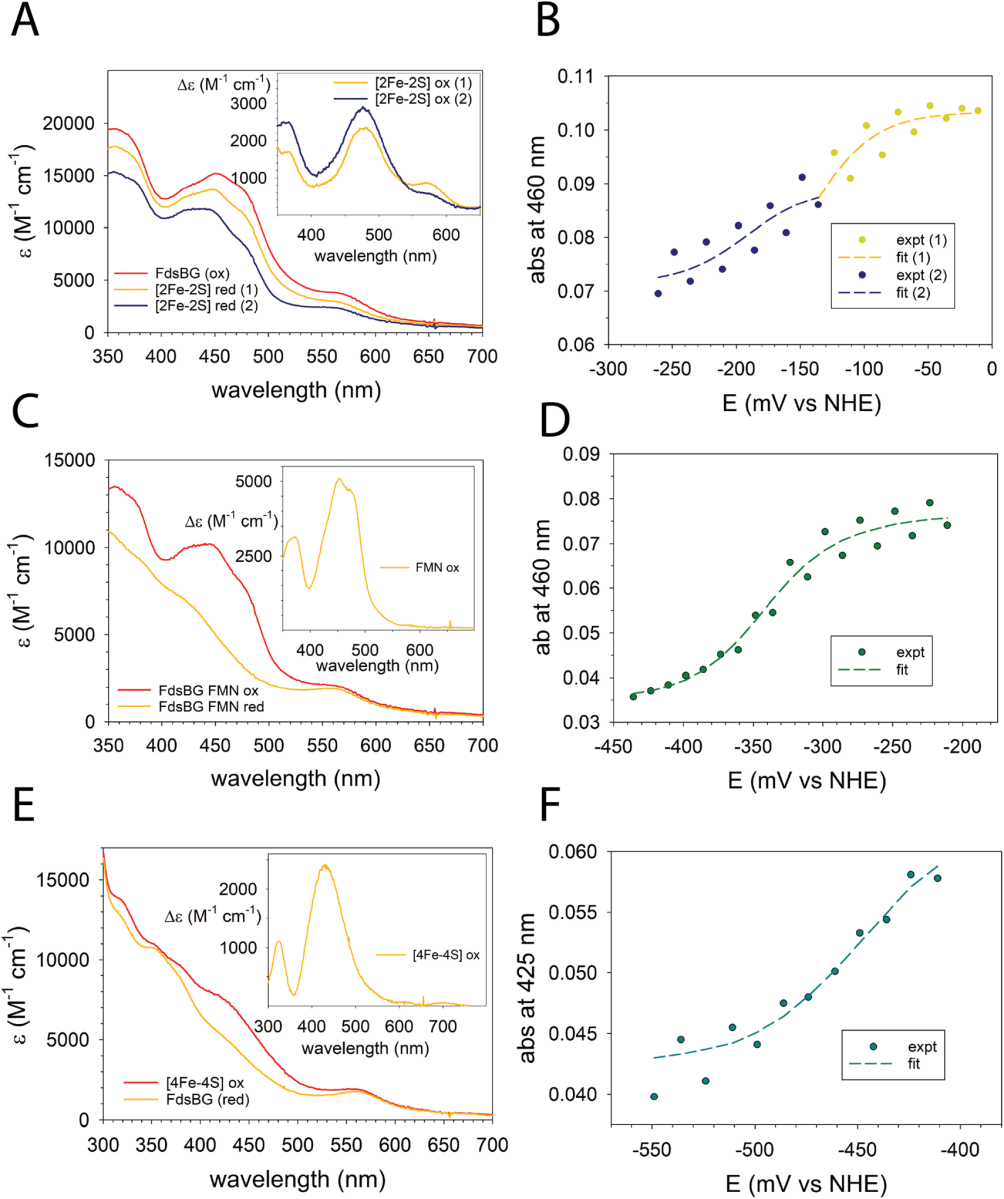
ReactLab redox spectral analysis for FdsBG (100 μM, pH 7) showing the (A) calculated spectra from the high potential region −0.26 < *E* < 0 V *vs.* NHE (red 1 and red 2 correspond to the one electron reduced flavo- and deflavo-FdsBG proteins); (B) calculated (broken lines) and experimental absorbances at 460 nm as a function of potential (*E*_1_ −0.11 V, *E*_2_ −0.19 V); (C) calculated spectra from the mid potential region −0.45 < *E* < −0.21 V; (D) calculated (broken line) and experimental absorbances at 460 nm as a function of potential (*E*_1_ −0.25, *E*_2_ −0.34 V); (E) calculated spectra from the low potential region −0.55 < *E* < −0.41 V; (F) calculated (broken line) and experimental absorbances at 425 nm as a function of potential (*E*_1_ −0.45 V). The mediators present (all 80 μM) are [Fe(*trans*-diammac)]^3+^, [Co(AMMEN_4_S_2_sar)]^3+^, [Co(AMMEN_5_Ssar)]^3+^, [Co(sep)]^3+^, [Co(AMMEsar)]^3+^, [Co(ClMeClAbsar)]^3+^ and [Co(*cis*-diammac)]^3+^. See ESI† for their chemical structures. The insets to panels (A, C and E) are the difference spectra calculated from the spectral data in each main panel.

The cofactor with most negative redox potential in FdsBG is the [4Fe–4S]^2+/+^ cluster, which has been characterised previously by UV-vis^27^ and EPR^24^ spectroscopy, while the [2Fe–2S]^2+/+^ cluster has the most positive potential. The closely spaced FMN/FMNH˙ and FMNH˙/FMNH^−^ redox potentials lie between those of the two Fe–S clusters. The transition from fully oxidised to fully (four-electron) reduced FdsBG theoretically involves five different UV-vis spectra and four redox potentials. A simultaneous global fit of so many parameters is not practical. The redox potentials of the three cofactors are sufficiently well separated that the entire set of potential-dependent spectra could be broken into three sections (here designated ‘low’, ‘mid’ and ‘high’ potential) and analysed individually as one- or two-electron steps. All potential-dependent spectra are shown in the ESI (Fig. S2A–C)† and the three partially overlapping sections are illustrated in a single wavelength plot (at 460 nm) as a function of potential and pH (Fig. S2D†). This plot shows the stepwise decrease in absorbance at 460 nm upon reduction which is then reversed on oxidation. Data at pH 7 are shown in Fig. 3A–F, while remaining raw data and spectral analyses are in the ESI (Fig. S3 and S4).†

The [2Fe–2S]^2+^ cluster in the FdsG subunit is reduced first in a single electron reaction ([2Fe–2S]^2+/+^). However, as noted previously,^24,27^ this region comprises two closely separated one-electron steps due to the [2Fe–2S]^2+/+^ clusters of FdsBG and its deflavo form (*i.e.* lacking FMN), which comprises 50% of the total protein sample. The potential-dependent spectra were separated into two regions and modelled as independent one-electron redox reactions (eqn (1)) with the truncated potential ranges (yellow and blue dots in Fig. 3B) avoiding spectral overlap from the two chromophores as much as possible. The spectra in this region (Fig. 3A) are dominated by transitions from the underlying oxidised FMN cofactor, so changes due to [2Fe–2S]^2+^ cluster reduction are more clearly appreciated by viewing their difference spectra (Fig. 3A, inset) which show the difference maxima (at 571, 479 and 364 nm) characteristic of the [2Fe–2S]^2+^ cluster, given that the spectrum of reduced [2Fe–2S]^+^ is featureless.^50–52^ The absence of the FMN cofactor in deflavo-FdsBG evidently influences the redox potential of the [2Fe–2S] cluster but an unambiguous assignment of the two [2Fe–2S]^2+/+^ couples to holo-FdsBG or deflavo-FdsBG is difficult. It is known from X-ray crystallography^24^ that there is a significant conformational change going from holo-FdsBG to deflavo-FdsBG especially involving residues Asp184 and Glu185 (Fig. 2). No significant pH dependence of these potentials was found (Table 1).

FMN reduction was modelled as consecutive one-electron reactions (eqn (2)) at each pH value for the two half-potentials FMN/FMNH˙ and FMNH˙/FMNH^−^. However, the two couples are in proximity so the semiquinone FMNH˙ is unstable with respect to disproportionation to the fully oxidised (FMN) and reduced (FMNH^−^) forms. The calculated spectrum of the flavin semiquinone contributes minimally due to its negligible accumulation at all potentials. Previously reported time-resolved stopped flow measurements allowed the UV-vis and EPR spectra of the metastable transient FMNH˙ form to be captured^24^ which decayed rapidly (within seconds). Under the equilibrium conditions employed here there was no possibility of observing the semiquinone radical. Across the potential range −250 to −400 mV the prominent maxima from the FMN chromophore essentially vanish (Fig. 3C). The difference spectrum over this region between the fully oxidised and reduced flavin (Fig. 3C, inset) highlights the FMN chromophore with its characteristic maxima at 452 and 375 nm typical of oxidised flavin cofactors.^53^ One notable feature is that the change in molar absorptivity (Fig. 3C, inset) is only ∼5000 M^−1^ cm^−1^, which is approximately half of the expected value for reduction of a flavin chromophore. As mentioned above, the protein mixture comprises *ca.* 50% deflavo-FdsBG which accounts for the smaller than usual change in spectrum. As protonation accompanies reduction of FMN, the midpoint potentials shift negatively as the pH rises, but the observed shifts (Table 1) are small and much less than expected for a 2e^−^/2H^+^ reaction (−59 mV pH^−1^); the data are more consistent with an overall 2e^−^/H^+^ reaction (−29.5 mV pH^−1^). The p*K*_a_ of FMNH˙ semiquinones in flavoproteins are typically too high to be measured potentiometrically^54^ so proton transfer accompanies the first reduction while the second electron transfer generates the monoanion.

For the [4Fe–4S]^2+^ cluster, a modest decrease in absorbance in the range 400–500 nm was apparent (Fig. 3E) upon electrochemical reduction, which is characteristic of a [4Fe–4S]^2+^ cluster.^55–57^ The difference spectrum (Fig. 3E, inset) accentuates this feature. Of the three chromophores present, the overall absorbance changes for the [4Fe–4S]^2+^ cluster were the smallest. A more challenging issue was that data collected at large negative potentials at pH 6 were compromised by hydrogen evolution (bubbles) in the spectral beam. For the remaining data, no significant change in the [4Fe–4S]^2+/+^ redox potential was observed between pH 7 and pH 8 (Table 1) including the published data at pH 7.5.^27^ Only a single potential-dependent spectral change was apparent so the [4Fe–4S]^2+/+^ potentials of the FdsBG holoprotein and its deflavo form must be very similar.

### Cyclic voltammetry

Electrochemical methods were applied in characterising the catalytic activity of the subcomplex FdsBG toward NAD^+^ reduction and NADH oxidation. For all experiments the concentration of FMN-containing FdsBG was used as the deflavo form cannot react with either NAD^+^ or NADH.

#### FdsBG-catalysed NAD^+^ reduction

FdsBG-catalysed NAD^+^ reduction was investigated using electrochemically reduced methyl viologen (MV^+^˙) as the electron donor. The pH dependence of FdsDABG formate dehydrogenases has been well studied with optimal activity for formate oxidation typically around pH 7.5.^19,42,58^ Additionally, MV^2+/+^˙ has been explored as a mediator supporting CO_2_ reductase activity for other formate dehydrogenase enzymes.^59,60^ In the present study, the substrate being reduced is NAD^+^ (rather than CO_2_) and the FMN cofactor in the FdsB subunit is the active site.

**Fig. 4:**
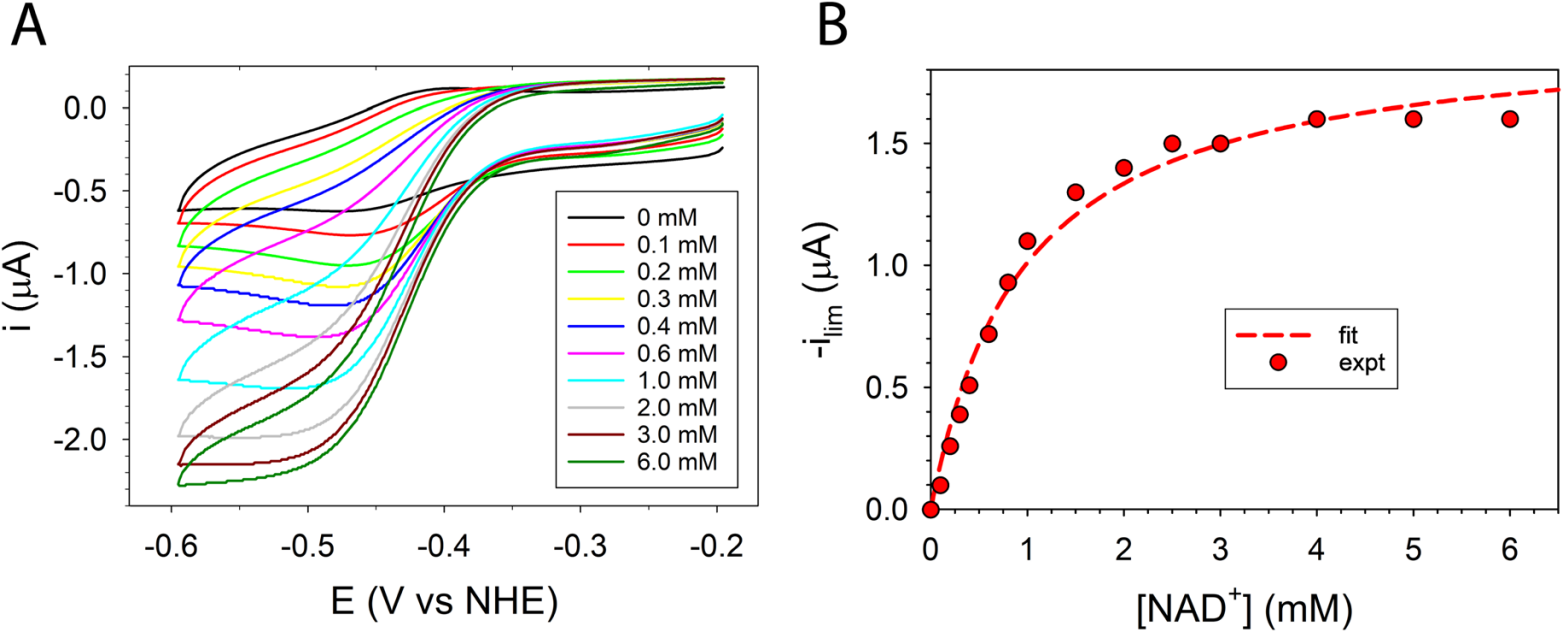
(A) CVs of methyl viologen (60 μM) in buffer (50 mM phosphate, pH 7.5) containing FdsBG (0.34 μM) at increasing concentrations of NAD^+^ (0–6 mM). (B) Plot of baseline subtracted electrocatalytic limiting cathodic current (μA at −0.55 V *vs.* NHE) against NAD^+^ concentration and fit to eqn (4) (*K*_M,NAD^+^_ = 1.2(1) mM).

The cyclic voltammetry response of MV^2+^ with FdsBG gave the expected single electron, quasi-reversible MV^2+/+^˙ redox response (*E*′ = −0.43 V *vs.* NHE) with a peak-to-peak separation of 70 mV and anodic/cathodic peak current ratio of unity (Fig. 4A, black curve). Upon the addition of NAD^+^, the symmetrical MV^2+/+^˙ response changed initially to an asymmetric waveform then eventually to a sigmoidal shape accompanied by an approximately five-fold increase in cathodic current (Fig. 4A). This change in shape is indicative of an electrocatalytic (EC_cat_) mechanism. At high concentrations of NAD^+^ (>4 mM) a steady state is reached where the rate of FdsBG reduction by MV^+^˙ is equal to the rate of NAD^+^ turnover by reduced FdsBG. The limiting catalytic peak current (*i*_lim_) at −0.55 V *vs.* NHE as a function of NAD^+^ concentration was modelled with eqn (4) and an apparent Michaelis constant was calculated (*K*_M,NAD^+^_ = 1.2(1) mM, Fig. 4B). Biochemical analysis of the highly homologous FdsBG (from *R. capsulatus*) found a similar value for NAD^+^ reduction (*K*_M,NAD^+^_ 1.1 mM).^25^ As a control, in the absence of FdsBG, no enhancement of MV^2+^ cathodic current is seen upon addition of NAD^+^ (Fig. S5†).

The less negative redox potential mediator safranin T (Fig. S1,†*E*′ = −0.29 V *vs.* NHE, pH 7) gave no change in current as a function of NAD^+^ concentration (data not shown). Given that the redox potential of the NAD^+^/NADH couple (−0.32 V, pH 7)^61^ is more negative than safranin T this was not unexpected. However, simple thermodynamic arguments alone do not predict the rates of outer sphere electron transfer between mediator and FdsBG. The mediators [Co(ClMeClAbsar)]^3+/2+^ (−0.45 V *vs.* NHE), [Co(*cis*-diammac)]^3+/2+^ (−0.50 V, Fig. S1†) were also tested as mediators but neither could support FdsBG-catalysed NAD^+^ reduction. Clearly this is not a thermodynamic constraint as [Co(ClMeClAbsar)]^2+^ and [Co(*cis*-diammac)]^2+^ are similarly strong reductants as MV^+^˙ (−0.43 V). Furthermore, these complexes in their Co^II^ state can reduce FdsBG as shown by UV-vis spectroelectrochemistry (Fig. 3 and ESI Fig. S2–S4†). In this case, the absence of a catalytic current is due to kinetic limitations where Co^II^-mediated reduction of FdsBG is too slow to sustain catalysis. The organic mediator MV^2+/+^˙ is flat and like NAD^+^/NADH may enter the binding pocket adjacent to the FMN cofactor for rapid electron transfer while the larger coordination complexes are hindered, and electron transfer is slowed significantly.

#### FdsBG-catalysed NADH oxidation

As mentioned, most metal-dependent formate dehydrogenases are bidirectional and can also catalyse CO_2_ reduction to formate. As the site of hydride exchange with NADH/NAD^+^ is still the FMN cofactor, this implies that the FsdBG subcomplex should also be bidirectional and biochemical studies on this enzyme^20,24^ and the highly homologous *R. capsulatus* FdsBG^25^ have supported this. Here, we investigated FdsBG NADH:O_2_ oxidoreductase (diaphorase) activity electrochemically but with mediators that have redox potentials more positive than the NAD^+^/NADH potential to bias the chemistry in favour of NADH oxidation. As above, control experiments were first explored to ensure that selected mediators did not oxidise NADH without FdsBG present. A range of common organic mediators (Fig. S1†) were tested including methylene blue (MB^+^) and phenazine methosulfate (PMS). In the absence of FdsBG, the reversible 2-electron response of PMS (Fig. S6†) showed an enhanced anodic current upon addition of NADH. This eliminated PMS as a practical mediator of FdsBG-catalysed NADH oxidation due to its indiscriminate oxidation activity.

Methylene blue was found to be the most suitable electron acceptor for FdsBG-catalysed NADH oxidation. Voltammetry of MB^+^ alone resulted in the expected quasi-reversible two-electron redox response (*E*′ = +0.010 V *vs.* NHE, pH 7) with a peak-to-peak separation of 70 mV (Fig. S7†). The addition of NADH (in excess) resulted in no significant increase in current although the waveform became less peak-shaped (flattened). Thermodynamically, NADH (*E*′ −0.33 V, pH 7) is capable of reducing MB^+^ (ref. 62 and 63) and bulk reduction of MB^+^ to its colourless leuco form (MBH) was observed upon addition of excess NADH to the electrochemical cell. However, this reaction is slow on the voltammetric timescale as no enhancement in MBH oxidation current was found when NADH was added (Fig. S7†). Upon addition of FdsBG the anodic current increased as anticipated (Fig. S7†).

**Fig. 5:**
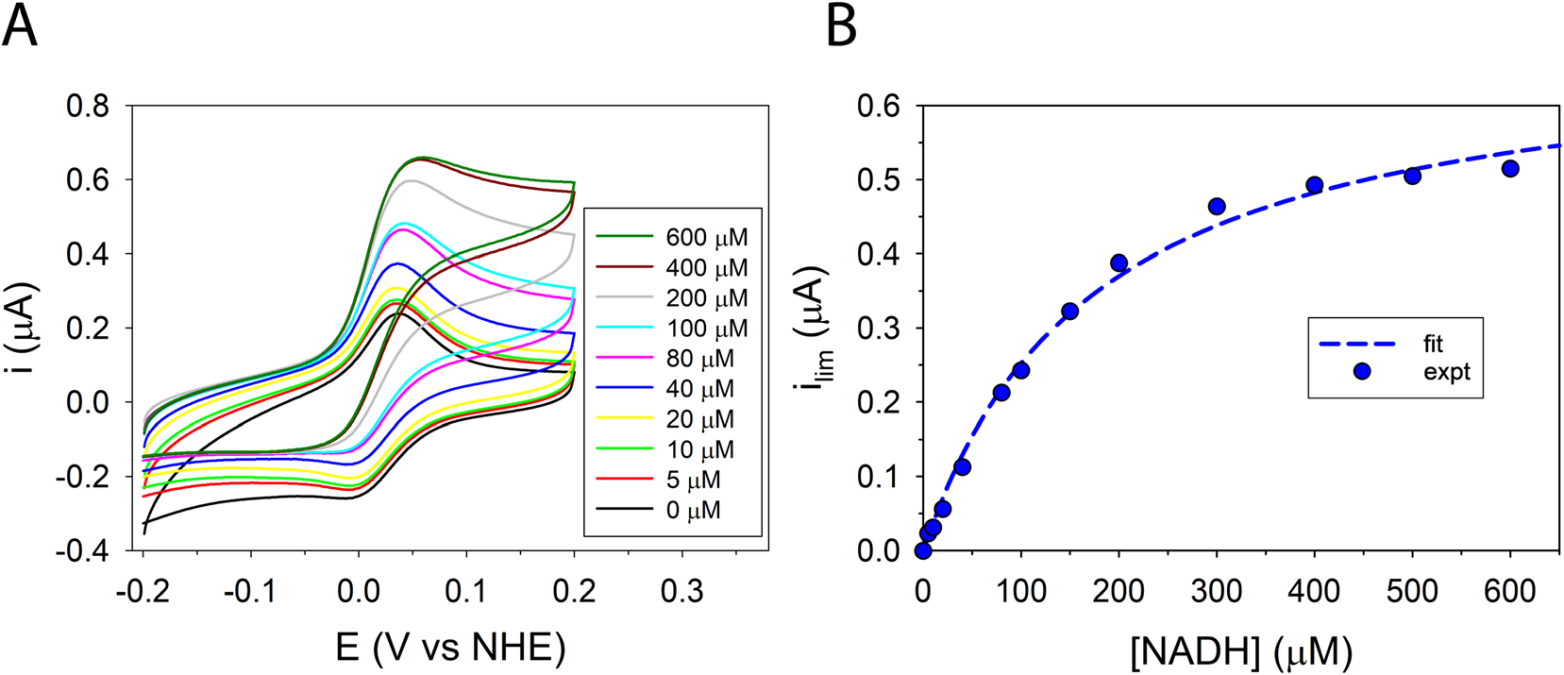
(A) Selected CVs of methylene blue (20 μM) (50 mM phosphate buffer, pH 7.5) containing FdsBG (0.34 μM) at increasing concentrations of NADH (0–600 μM). (B) Plot of baseline subtracted electrocatalytic limiting cathodic current (μA at +0.15 V *vs.* NHE) against NADH concentration and fit to eqn (4) (*K*_M,NADH_ = 1.7(1) × 10^2^ μM).

In the presence of FdsBG, the CV waveform of MB^+^ as a function of NADH concentration changed from symmetrical to the typical asymmetric catalytic shape paired with a significant increase in anodic current (Fig. 5A). At approximately 400 μM NADH, the catalytic anodic current reached saturation as expected for an enzyme-catalysed reaction and the cathodic peak of the wave vanished. The limiting catalytic peak current (*i*_lim_), corrected for the response of the mediator in the absence of NADH, was measured at +0.15 V *vs.* NHE as a function of NADH concentration (Fig. 5B), giving *K*_M,NADH_ = 1.7(1) × 10^2^ μM, (pH 7.5). Comparable biochemical data from the holoenzyme FdsDABG and the FdsBG subcomplex (*C. necator*) for NADH oxidation have been published with *K*_M,NADH_ (or *K*_d,NADH_) values of 46 μM^22^ and 1.9 × 10^2^ μM,^24^ respectively. Data published for FdsBG (from *R. capsulatus*) reported a similar value of *K*_M,NADH_ 1.3 × 10^2^ μM.^25^

#### pH dependence

**Fig. 6:**
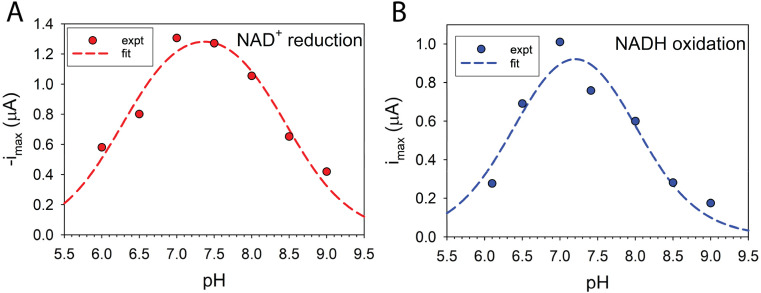
pH dependence of FdsBG (0.34 μM) catalysed (A) NAD^+^ (5 mM) reduction mediated by methyl viologen (60 μM) the broken curve is obtained from a fit to the experimental data using eqn (5) (p*K*_a1_ = 8.4(1), p*K*_a2_ = 6.3(1)) and (B) NADH (500 μM) oxidation mediated by methylene blue (60 μM) the broken curve is obtained from a fit to the experimental data using eqn (5) (p*K*_a1_ = 7.9(2), p*K*_a2_ = 6.5(2)). All CV data were collected at a scan rate of 5 mV s^−1^. The voltammograms are shown in the ESI (Fig. S8).†

The pH dependence of MV-mediated FdsBG-catalysed NAD^+^ reduction was explored over the range pH 6–9. The catalytic current (at saturating NAD^+^ concentration) exhibited a bell-shaped profile (Fig. 6A) with a maximum at approximately pH 7.5. Application of eqn (5) yielded p*K*_a_ values of 8.4(1) and 6.3(1) (Fig. 6A). The pH-dependence of MB-mediated FdsBG-catalysed NADH oxidation (Fig. 6B) showed remarkably similar behaviour, with protonation constants (7.9(2) and 6.5(2)) that are not significantly different from the NAD^+^ reduction profile. All raw CV data are available in the ESI (Fig. S8).† These values match previously reported pH profiles for FdsDABG-catalysed formate oxidation and CO_2_ reduction.^19,22,42,58^

The similarity of the profiles in Fig. 6A and B and the same p*K*_a_ values found in the FdsDABG holoenzyme imply that acid–base reactions at the NAD^+^/NADH binding FMN site, common to both enzymes, underpin the pH dependence of both FdsBG and FdsDABG catalysis. As shown by the spectroelectrochemistry results (Table 1) the redox potentials of the [4Fe–4S] cluster and [2Fe–2S] cluster are almost pH-independent while the FMN potential shifts slightly more negative at higher pH. The redox potential of MV^2+/+^˙ is pH independent, so the electrochemical driving force of the reductant does not change with pH for the system in Fig. 6A. The redox properties of the two-electron oxidant/reductant MB^+^/MBH are pH-dependent but not straightforward;^64^ in the range 6 < pH < 9 the dependence is −29 mV pH^−1^ (ref. 65) which equates to a 2e^−^/1H^+^ reaction in accord with the monocation MB^+^ and neutral MBH forms being the redox active species.

The changes in cofactor and mediator redox potentials with pH are small and do not correlate with the data in Fig. 6A and B. The substrate NAD^+^/NADH redox potential (*E*′ −0.32 V *vs.* NHE at pH 7) is pH-dependent with a shift of −29 mV pH^−1^ unit.^54^ At high pH, reduction of NAD^+^ becomes thermodynamically and kinetically less favoured which may contribute to the drop in current on the basic limb of the profile but this does not explain the same trend apparent in the NADH oxidation profile as the MB^+^/MBH redox potential is always much more positive than the FMN/FMNH^−^ (or NAD^+^/NADH) potential. Regarding the acidic limb of Fig. 6A, the X-ray crystal structure of FdsBG in complex with NADH (and in its absence) highlight several potential bases that may be involved in this modulation of activity. Two residues of interest are Asp184 and Glu185 (Fig. 2) which are close to the FMN group and form H-bonds (in their deprotonated forms) with the nicotinamide and/or FMN cofactor and protonation of these may disrupt the active site and weaken substrate binding. A more definitive explanation of the basic limbs of Fig. 6A and B remains the subject of further work.

#### Electrochemical simulations

Electrochemical simulations have been widely employed to understand biochemical kinetic and mechanistic properties of a redox active catalytic system.^43,66–69^ The objective is to model all experimental CV data based on a given mechanism that involve homogeneous chemical reactions and both homogeneous and heterogeneous electron transfer steps. The correct rate and equilibrium constants associated with these steps, the mediator redox potentials and heterogeneous electron transfer kinetics will reproduce all CV data over a range of substrate concentrations and sweep rates. The models used to simulate MV-mediated FdsBG-catalysed NAD^+^ reduction (Scheme 1A) and MB-mediated FdsBG-catalysed NADH oxidation (Scheme 1B) define all kinetic parameters.

**Fig. 7:**
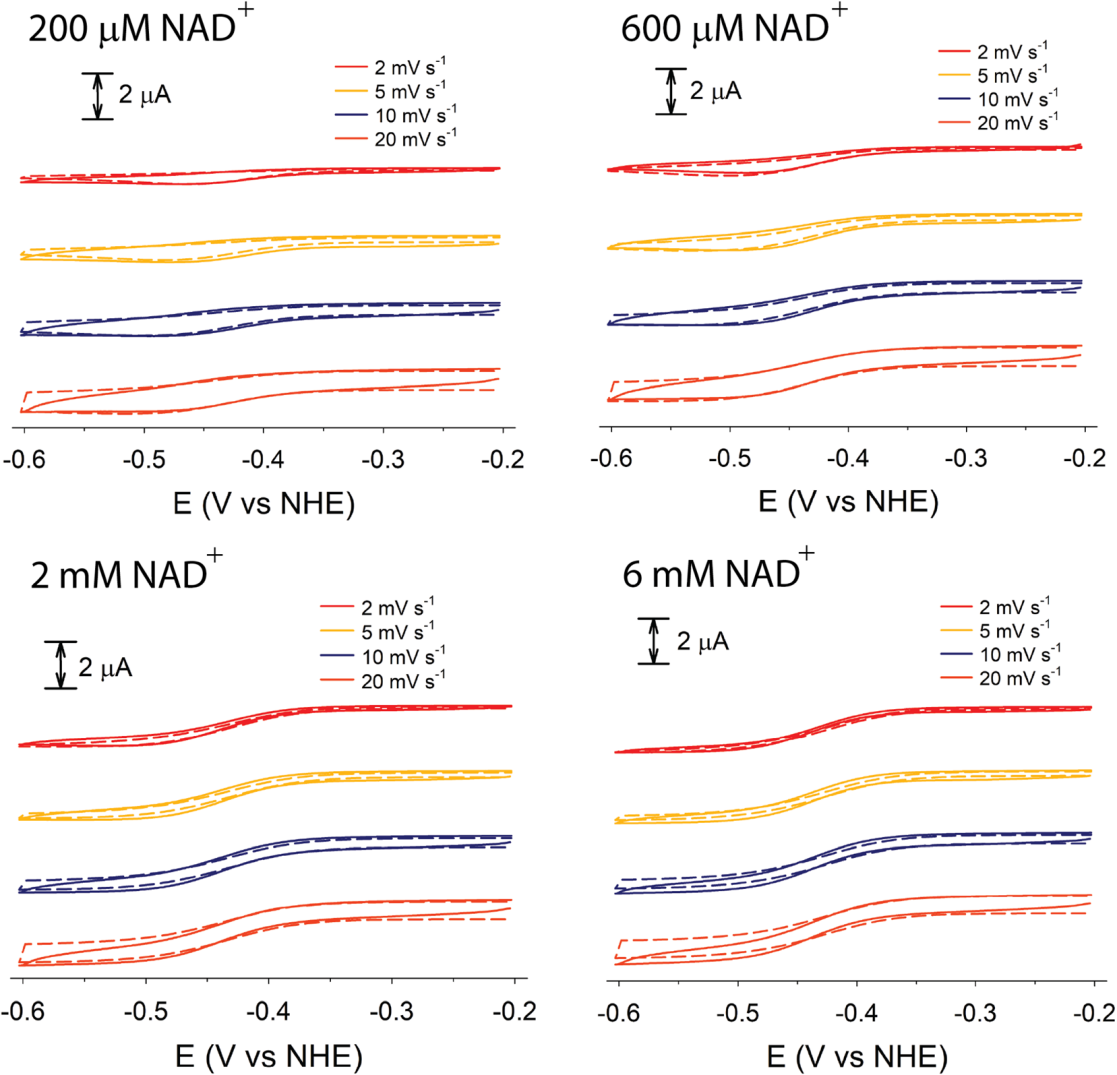
Experimental (solid lines) and simulated (broken lines) cyclic voltammograms of methyl viologen (60 μM) mediated FdsBG (0.34 μM) catalysed NAD^+^ reduction (concentrations shown) at different scan rates (50 mM phosphate buffer, pH 7.5).

**Fig. 8:**
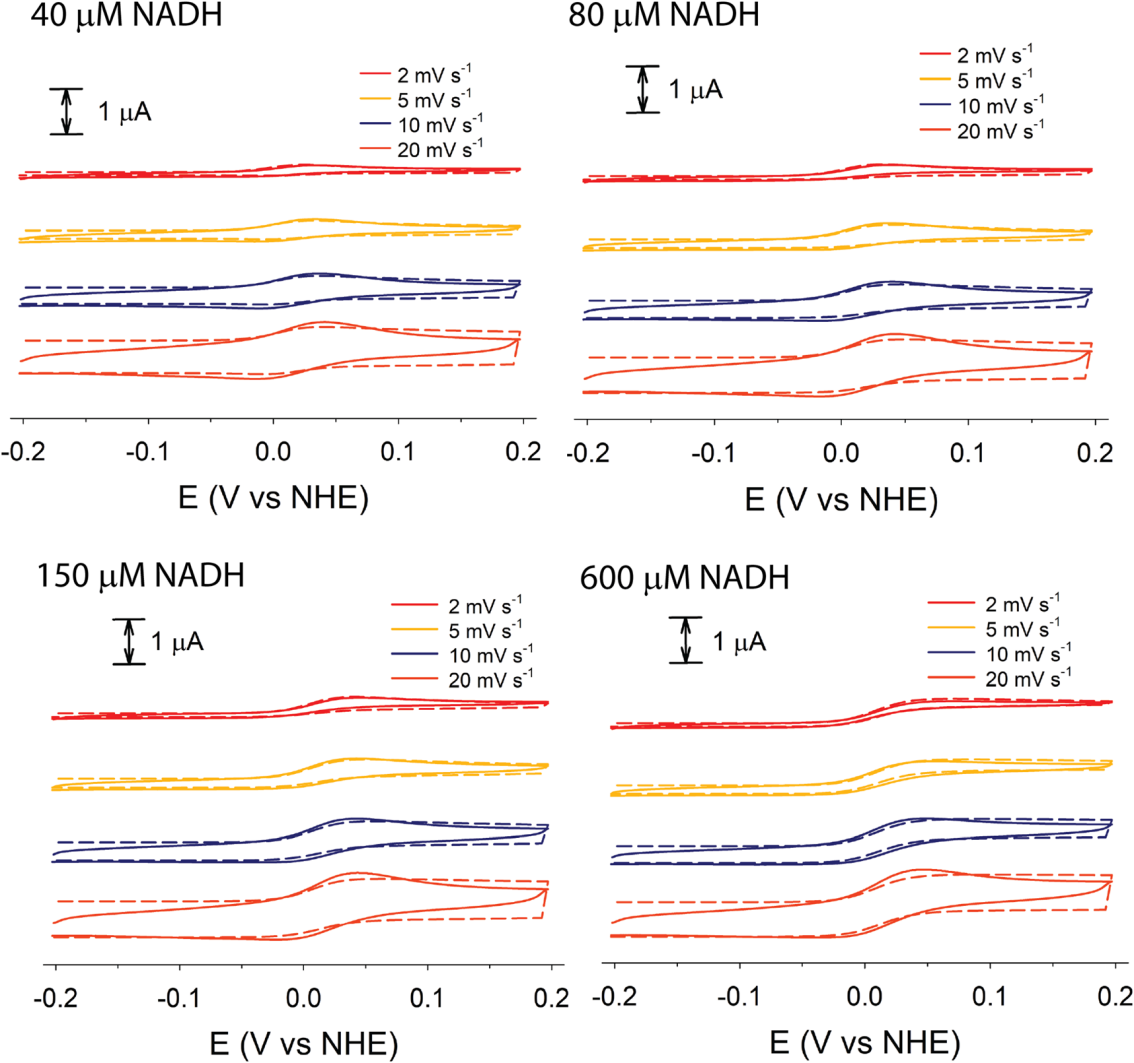
Experimental (solid lines) simulated (broken lines) cyclic voltammograms of methylene blue (20 μM) mediated FdsBG (0.34 μM) catalysed NADH oxidation (concentrations shown) at different scan rates (50 mM phosphate buffer, pH 7.5).

Although electrochemical analysis of electrode-confined (adsorbed) enzymes can also provide useful kinetic information,^42,66–69^ an advantage of the present approach with all species in solution is that accurate enzyme concentrations are known, although consumption of enzyme can be significantly greater than experiments using electrode-confined enzyme. For FdsBG electrocatalysis experiments, the simulations were performed at multiple scan rates (2, 5, 10 and 20 mV s^−1^) and substrate concentrations (NAD^+^: 0–6 mM, NADH: 0–600 μM) guided by the data in Fig. 4 and 5. The rate constants defined in Scheme 1 and assembled in Table 2 reproduced the experimental voltammetry profiles across a range of scan rates, and substrate concentrations (Fig. 7 and 8).

Experimental and simulated CV data of MV^2+^ (60 μM), FdsBG (0.34 μM) and NAD^+^ (200 μM to 6 mM) at different scan rates are shown in Fig. 7. The theoretical aspects underpinning the changes in waveform in an enzyme-catalysed electrochemical system as a function of substrate concentration and scan rate are well described elsewhere^70^ and also applied to synthetic (non-enzymatic) electrocatalysis where they are conveniently graphically represented by so-called kinetic zone diagrams.^44,71,72^ In this case, competition between the enzyme–substrate reaction (a function of substrate concentration) and the enzyme–mediator reaction is the major cause of the changes in waveform here. Variations in scan rate can also affect the CV profile but in the systems studied here the scan rates were deliberately confined to a narrow range to avoid excessive charging currents relative to the faradaic currents at micromolar concentrations. All CVs fall into the so-called ‘pure kinetic zone’ with subtle changes in wave symmetry and shape attributable to substrate concentrations.

At low NAD^+^ concentrations (200–600 μM), asymmetric peak-shaped curves are found where NAD^+^ mass transport limits the cathodic current. As the NAD^+^ concentration reaches saturating levels (∼6 mM) sigmoidal waveforms emerge characteristic of an electrochemical steady state. The CVs under these conditions are essentially independent of scan rate as the rate of the FdsBG_red_:NAD^+^ reaction is at its maximum (limited by the turnover number). At faster scan rates (20 mV s^−1^) and low NAD^+^ concentrations (200 μM), production of MV^+^˙ at the electrode exceeds its rate of consumption by NAD^+^-oxidised FdsBG and an anodic peak due to unreacted MV^+^˙ reappears.

The CV data of MB^+^ (20 μM) in the presence of FdsBG (0.34 μM) and NADH (40–600 μM) at different scan rates are also shown (Fig. 8). Again, at slower scan rates (2, 5 mV s^−1^) and low NADH concentrations (<100 μM), an asymmetric waveform is observed indicative of substrate limited currents where NADH depletion from the reaction layer attenuates catalysis. At higher NADH concentrations, the observed and simulated waveforms are more symmetrical and sigmoidal in shape although NADH depletion is still apparent in all CVs in Fig. 8 from the peak-shaped anodic profiles which is indicative of slower substrate turnover.

The CV waveforms are also sensitive to the electron transfer stoichiometry. The two-electron MB^+^/MBH redox reaction shows noticeably steeper CV traces (Fig. 8) than the single-electron MV^2+/+^˙ couple (Fig. 7) and this translates into similarly shaped catalytic profiles as expected.^40^

### Kinetic analysis

The overall kinetics of both the reduction of NAD^+^ and oxidation of NADH as catalysed by FdsBG conform to a two-substrate ping-pong mechanism as expected, with the enzyme alternating between reduced and oxidised forms. In principle, each catalytic cycle of the enzyme should consist of four steps: (i) substrate binding; (ii) turnover; (iii) product release and (iv) outer sphere electron transfer with the mediator to regenerate the active form of FdsBG (either reduced or oxidised). However, as will be shown, the unimolecular steps (ii) and (iii) are inseparable from (i) at practical experimental concentrations and scan rates and they therefore cannot be fitted independently within the same model. The scan rate and substrate concentration dependent data were modelled with a simpler scheme where substrate binding, turnover and product release are incorporated into a single bimolecular rate constant (*k*_red_ for NAD^+^ reduction or *k*_ox_ for the reverse NADH oxidation). Accurate rate constants describing enzyme reaction (*k*_ox_ and *k*_red_, Table 2) emerge at low substrate concentrations (so-called pure kinetic conditions or zone K)^71^ where substrate binding limits the catalytic current. The outer sphere electron transfer rate constants *k*_3_/*k*_4_ (for MV-driven NAD^+^ reduction) and *k*_5_/*k*_6_ (for MB-driven NADH oxidation) are accurately determined when the enzyme is saturated with substrate which give essentially sigmoidal profiles (within the zone KS).^71^ It is relevant that reduction of FdsBG by MV^+^˙ (*k*_3_) and oxidation of FdsBG by MB^+^ (*k*_5_) are effectively irreversible reactions due to the large driving force involved, so the reverse outer sphere electron transfer rate constants (*k*_4_ and *k*_6_) are uncertain, and their values have no influence on the simulations. Also the rates of the consecutive one-electron MV^+^˙ reductions of FdsBG_ox_ and FdsBG_int_ (*k*_3_, Scheme 1) were assumed to be equal based on their very similar driving forces.

**Scheme 2:**
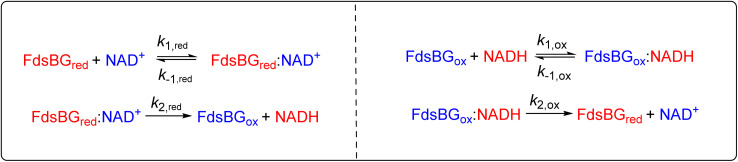
Substrate binding and turnover steps for NAD^+^ reduction and NADH oxidation by FdsBG. The rate constants are elaborated in eqn (7)–(10) below.

The mediator-independent rate constants *k*_ox_ and *k*_red_ obtained from the simulations are the same at 2.0 × 10^6^ M^−1^ s^−1^, so the equilibrium constant for the reaction is unity. This is consistent with the midpoint potentials of the FMN cofactor (−0.34 V *vs.* NHE at pH 7.5)^27^ and the NAD^+^/NADH couple (−0.33 V at pH 7.5) being the same within experimental error, meaning that the reaction is not biased in either direction.^61^ However, the data in Fig. 4 and 5 reveal *K*_M,NAD^+^_ and *K*_M,NADH_ values that differ by an order of magnitude. Following a typical Briggs–Haldane mechanism,^73^ the rate constants *k*_red_ and *k*_ox_ are composites of substrate binding and turnover (Scheme 2) as expressed in eqn (7) and (8), respectively.7
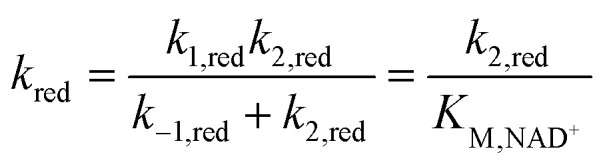
8
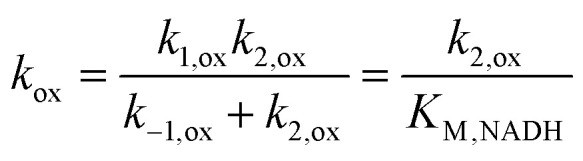
9
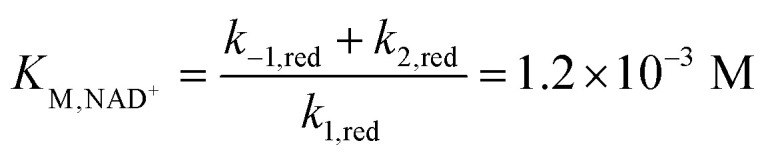
10
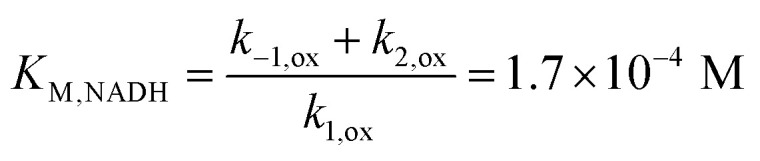


Substituting the experimental values for *K*_M,NAD^+^_ (eqn (9)) and *K*_M,NADH_ (eqn (10)) (see Fig. 4 and 5) and simulated *k*_ox_/*k*_red_ values (Table 2) into eqn (7) and (8) generates turnover numbers of *k*_2,red_ (NAD^+^ reduction) = 2.4 × 10^3^ s^−1^ and *k*_2,ox_ (NADH oxidation) = 3.4 × 10^2^ s^−1^. Pre-steady state kinetic investigations of FdsBG (*C. necator*)^24^ revealed a limiting rate of NADH oxidation (6.8 × 10^2^ s^−1^ at 278 K) which greatly exceeded the corresponding rate of formate oxidation at the Mo active site by the FdsDABG holoenzyme.^24^ Also of note are the large outer sphere electron transfer rate constants of FdsBG_ox_ with MV^+^˙ (*k*_3_) and FdsBG_red_ with MB^+^ (*k*_5_) showing MV^+^˙ and MB^+^ to be ideal artificial electron transfer partners. Electrochemical simulations with lower values for these rate constants could not produce the magnitudes of catalytic currents in Fig. 7 and 8 at saturating substrate concentrations regardless of substrate oxidation/reduction kinetics (*k*_ox_ and *k*_red_).

## Conclusions

This study has explored the redox properties and catalytic activity of the formate dehydrogenase subcomplex FdsBG including nicotinamide cofactor turnover, pH dependence, and optical spectroelectrochemistry, demonstrating how electrochemistry and simulations can be utilised to ascertain previously unknown kinetic properties of a NADH dehydrogenase enzyme. FdsBG provides an excellent enzymatic platform to explore the characteristics of both NADH oxidation and NAD^+^ reduction within the same system. The pH dependence of both the FdsBG redox potentials and NAD^+^/NADH turnover have been obtained (6 < pH < 8), augmenting previously reported electrochemical^27^ and biochemical^19,24^ data. To the best of our knowledge this is the first example of a full electrochemical kinetic analysis of a reversible NADH dehydrogenase system. The parameters show that FdsBG is a highly active bidirectional catalyst of NAD^+^/NADH turnover and also that MV^+^˙ and MB^+^ are highly effective artificial electron partners to sustain catalysis and demonstrate the effectiveness of FdsBG in catalysing the electrochemical regeneration and interconversion of NAD^+^ and NADH. Opportunities to incorporate FdsBG as an electrochemically-driven generator of either NADH or NAD^+^ can now be considered coupled to other NAD^+^/NADH-dependent enzymes.

## Data availability

Additional data supporting this article have been included as part of the ESI.†

## Author contributions

PDG: data curation, formal analysis, investigation, methodology, validation, writing – original draft, writing – review & editing. DN: resources – protein purification, writing – review & editing. SH: resources – protein purification, writing – review & editing. RH: funding acquisition, writing – review & editing. PVB: conceptualization, data curation, formal analysis, funding acquisition, methodology, project administration, resources, software, validation, visualization, writing – original draft, writing – review & editing.

## Conflicts of interest

There are no conflicts to declare.

## Supplementary Material

SC-OLF-D5SC00570A-s001
