# Supplementary material for: Reversible enzyme-catalysed NAD+/NADH electrochemistry
Source: Chem Sci. 2025 Mar 4;16(14):6035–49. doi: 10.1039/d5sc00570a (PMC11891784; doi:10.1039/d5sc00570a)
Supplement: SC-016-D5SC00570A-s001 [file SC-016-D5SC00570A-s001.pdf]

Supplementary Information (SI) for Chemical Science.
This journal is © The Royal Society of Chemistry 2025

Supplementary Information

Reversible Enzyme-Catalysed NAD^+/NADH Electrochemistry

Peter D. Giang,[a] Dimitri Niks,[b] Sheron Hakopian,[b] Russ Hille,[b] Paul V. Bernhardt[a*]

[a] School of Chemistry and Molecular Biosciences, University of Queensland, Brisbane 4072, Australia

[b] Department of Biochemistry, University of California, Riverside, USA

*p.bernhardt@uq.edu.au

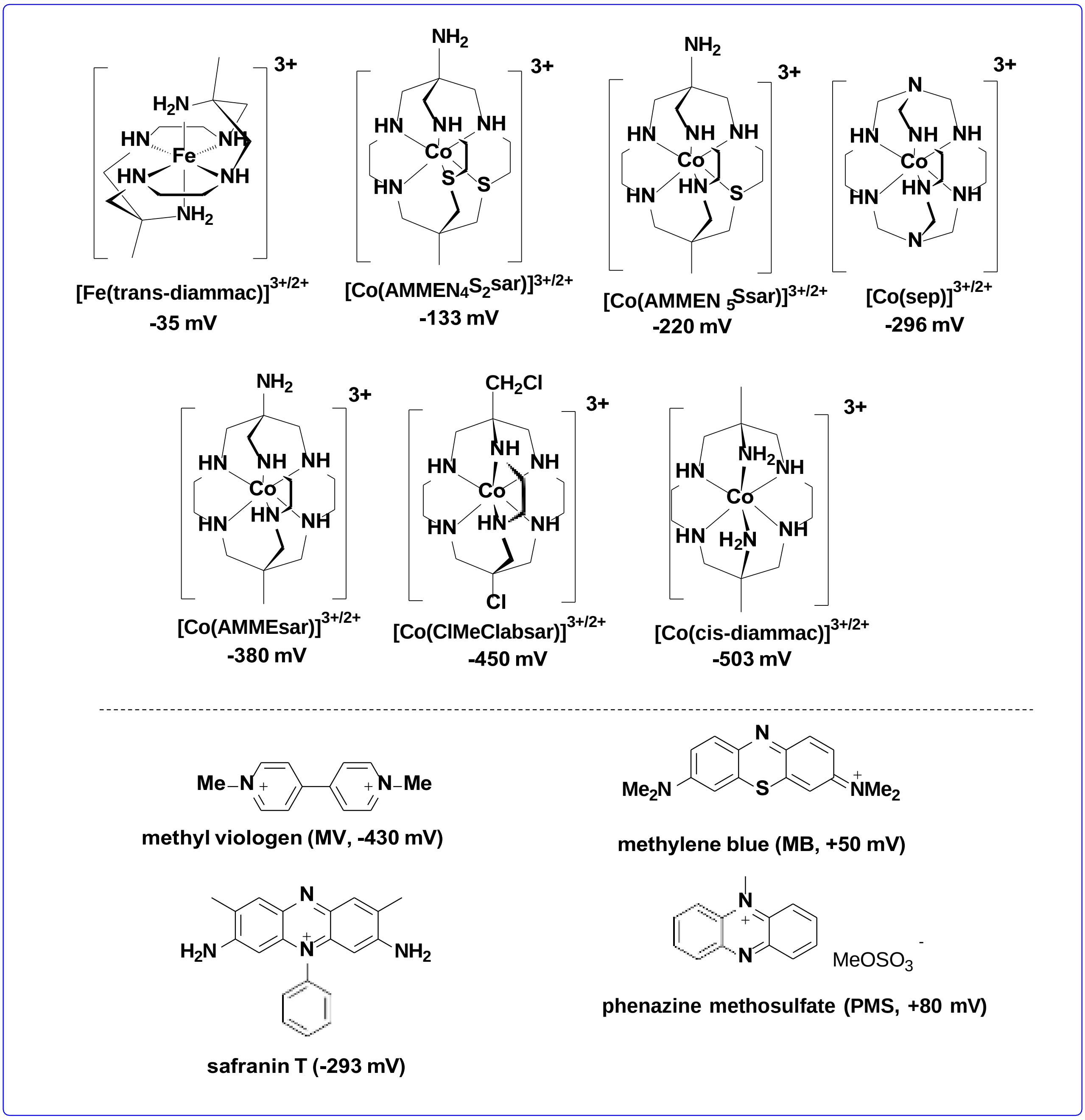

Figure S1. Chemical structures of all mediators used in this work and their approximate redox potentials vs NHE at pH 7.

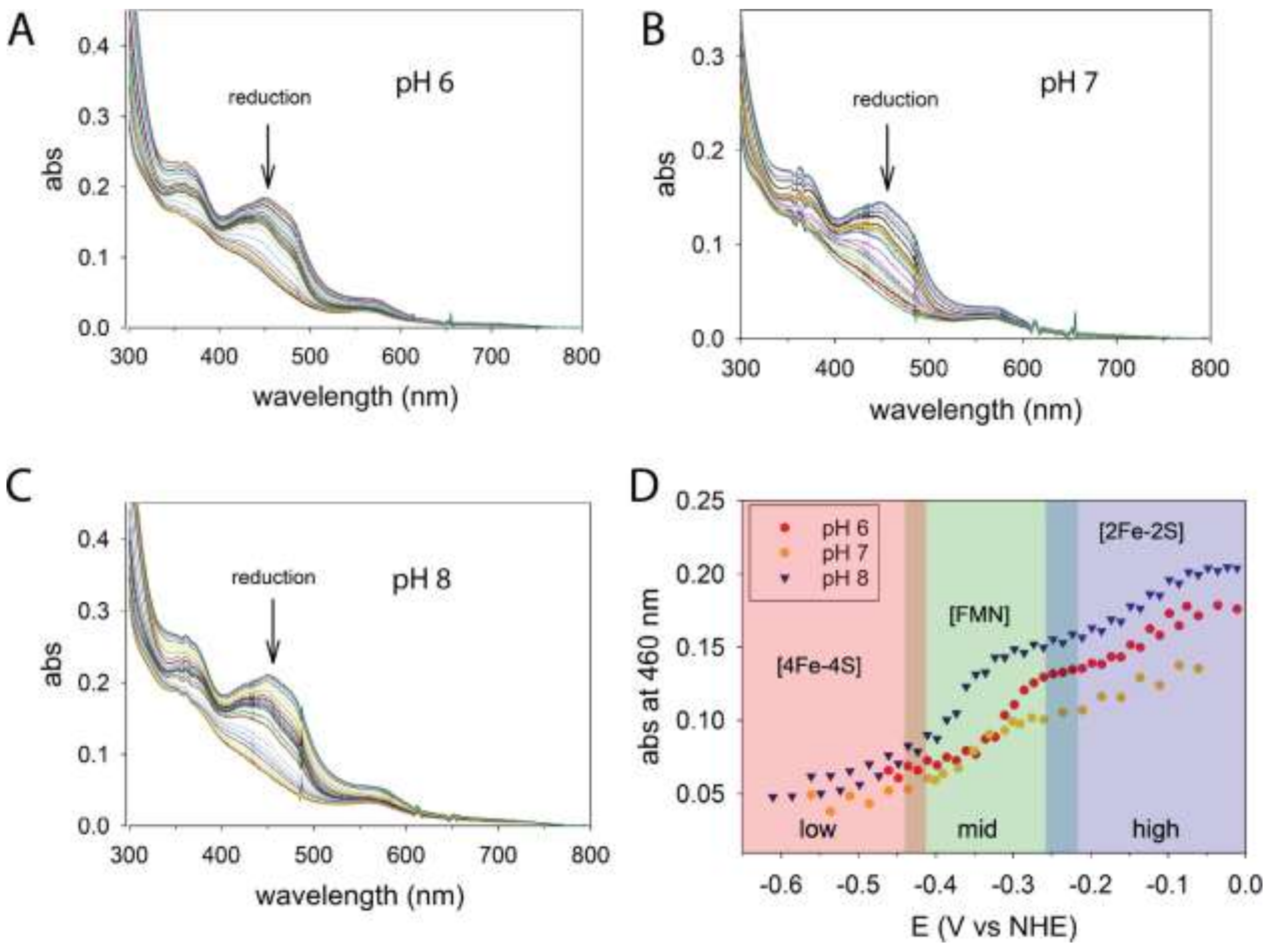

Figure S2. Optical spectroelectrochemistry data for FdsBG (100 μM) at (A) pH 6 (−0.45 < *E* < 0 V vs NHE); (B) pH 7 (−0.55 < *E* < −0.05 V vs NHE) and (C) pH 8 (−0.55 < *E* < 0 V vs NHE); (D) absorbance changes at 460 nm as a function of potential and pH indicating the three data regions that were fit independently.

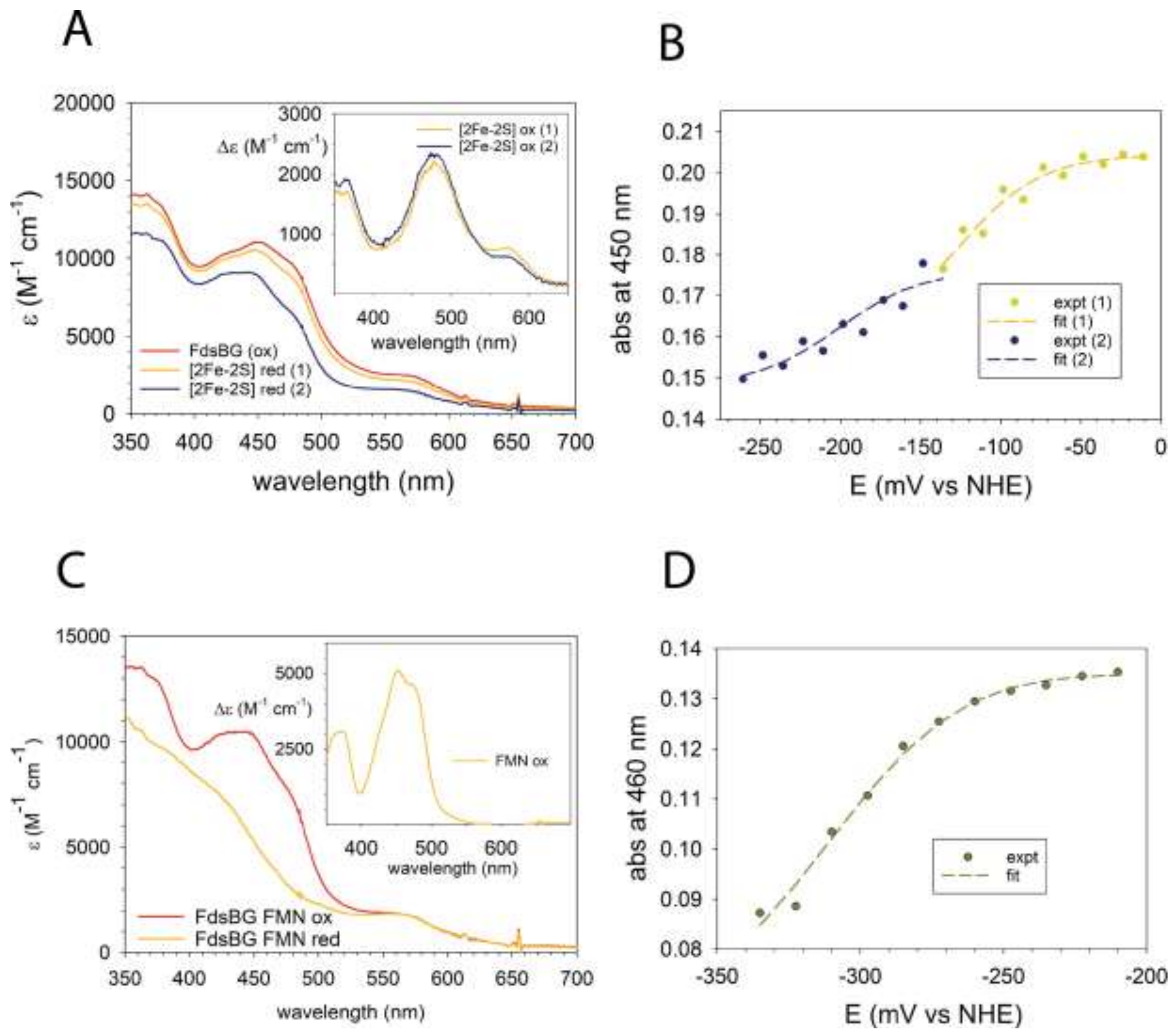

Figure S3. Reactlab Redox spectral analysis for FdsBG (100 µM, pH 6) showing the (A) high potential region −210 < *E* < 0 mV vs NHE (difference spectra shown in the inset); (B) calculated and experimental absorbances at 450 nm as a function of potential (E1 −120, E2 −150 mV); (C) mid potential region −335 < *E* < −210 mV (difference spectrum shown in the inset); (D) calculated and experimental absorbances at 460 nm as a function of potential (E1 −240, E2 −310 mV). Difference spectra are shown in the insets of panels A and C.

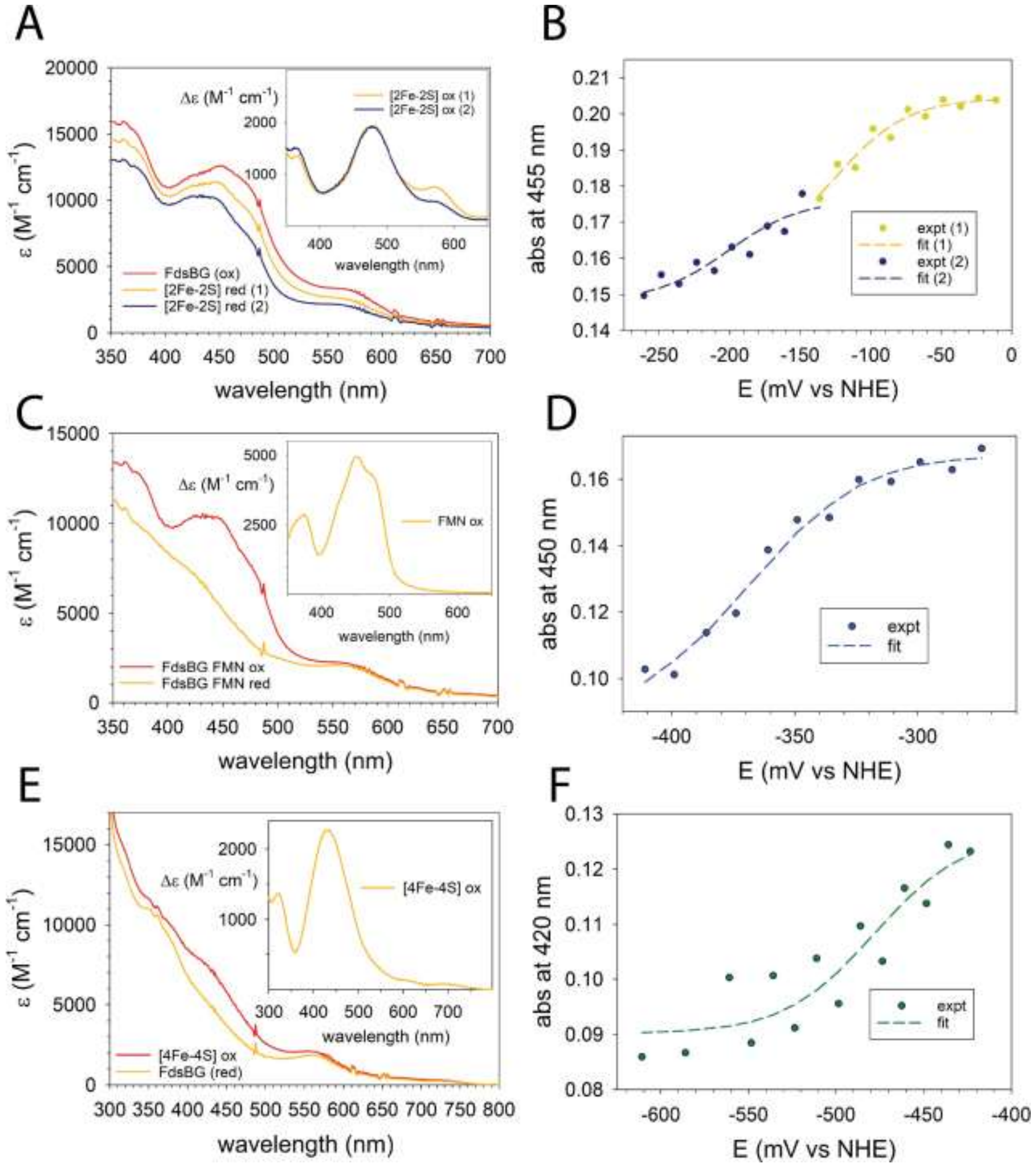

Figure S4. Reactlab Redox spectral analysis for FdsBG (100 µM, pH 8) showing the (A) high potential region −260 < *E* < −10 mV vs NHE; (B) calculated and experimental absorbances at 455 nm as a function of potential (E1 −130, E2 −200 mV); (C) mid-potential range −410 < *E* < −260 mV; (D) calculated and experimental absorbances at 450 nm as a function of potential (E1 −320, E2 −370 mV); (E) low potential range −610 < *E* < −420 mV; (F) calculated and experimental absorbances at 420 nm as a function of potential (E1 −480 mV). Difference spectra are shown in the insets of panels A, C and E.

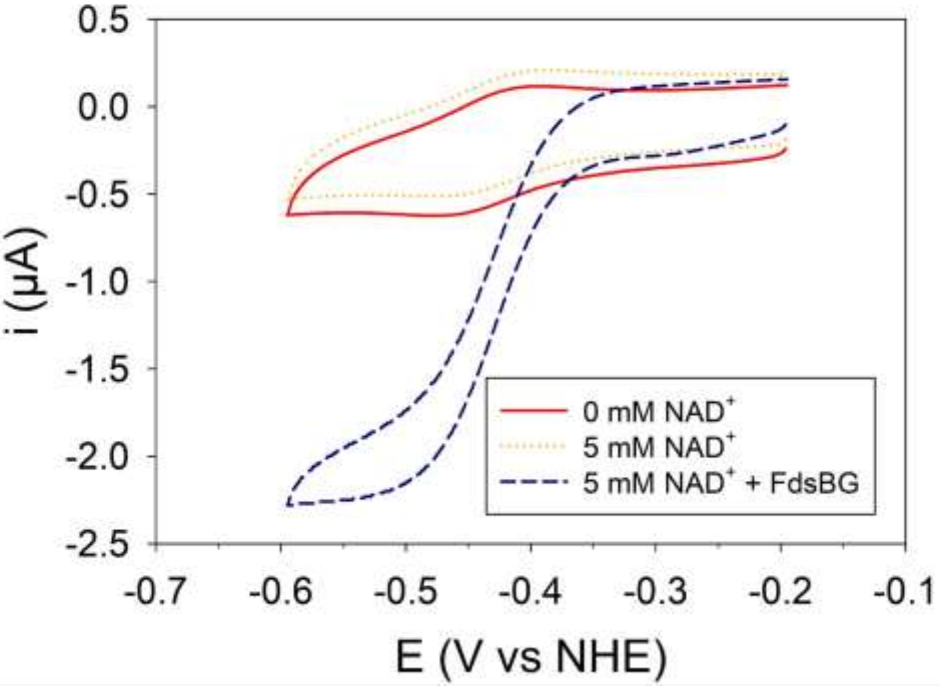

Figure S5. Cyclic voltammetry of methyl viologen (60 µM) (red curve); methyl viologen (60 µM) plus NAD^+ (5 mM) (yellow curve) and methyl viologen (60 µM) plus NAD^+ (5 mM) and FdsBG (0.34 µM) (blue curve). Scan rate 5 mV s^{-1}, 50 mM phosphate buffer, pH 7.5.

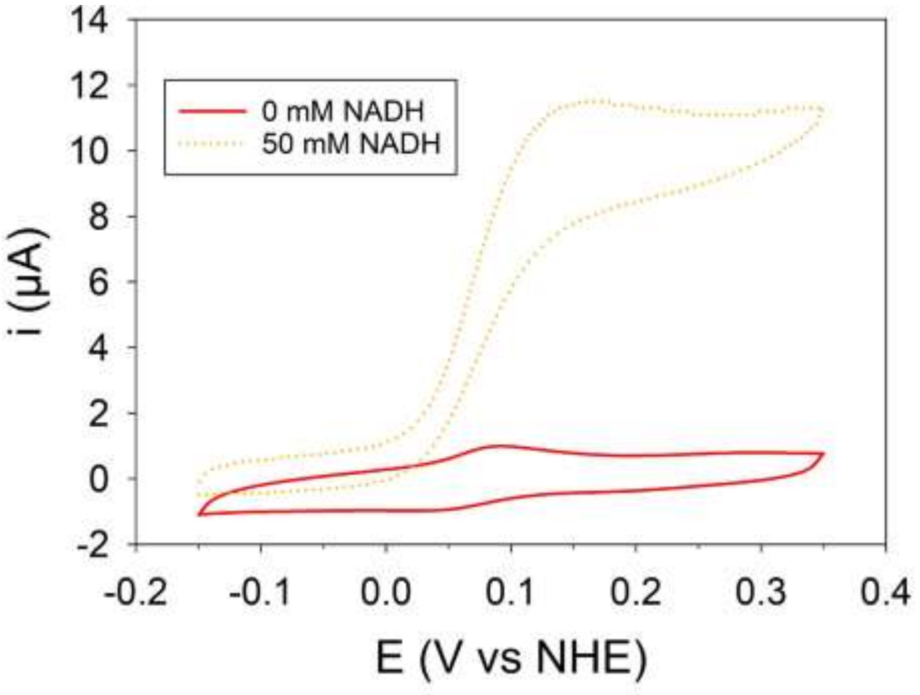

Figure S6. Cyclic voltammetry of phenazine methosulfate (40 µM) (red curve) and phenazine methosulfate (40 µM) plus NADH (50 mM) (yellow curve). Scan rate 5 mV s^{-1}, 50 mM phosphate buffer, pH 7.5.

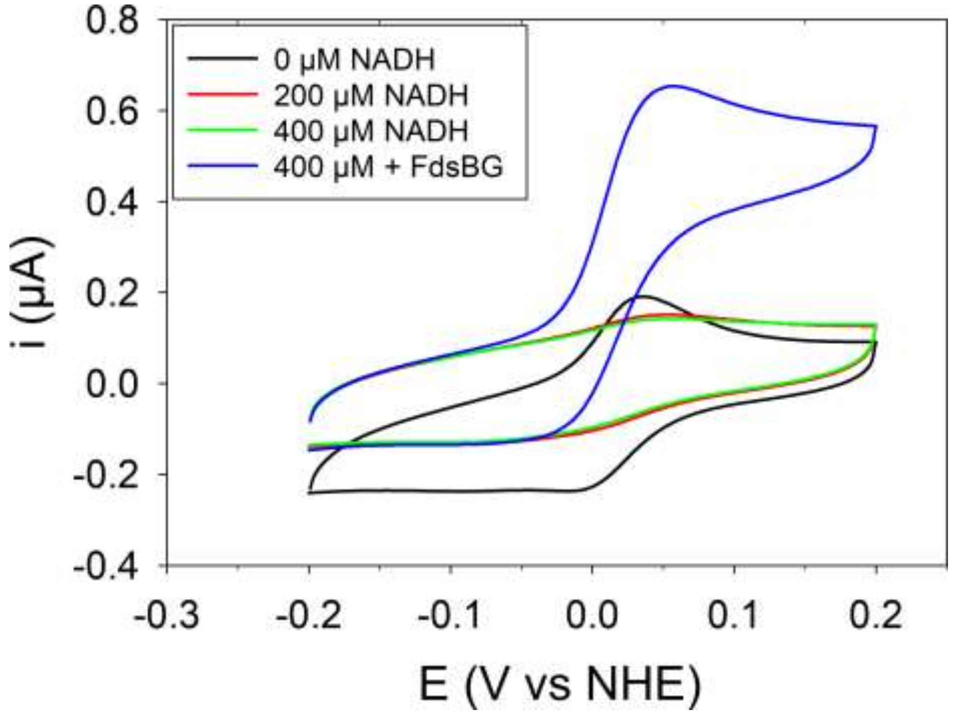

Figure S7. Cyclic voltammetry of methylene blue (20 µM) (black curve); methylene blue (20 µM) plus NADH (200 mM) (red curve); methylene blue (20 µM) plus NADH (400 mM) (green curve); methylene blue (20 µM) plus NADH (400 mM) plus FdsBG (0.34 µM, blue curve). Scan rate 5 mV s^{-1}, 50 mM phosphate buffer, pH 7.5.

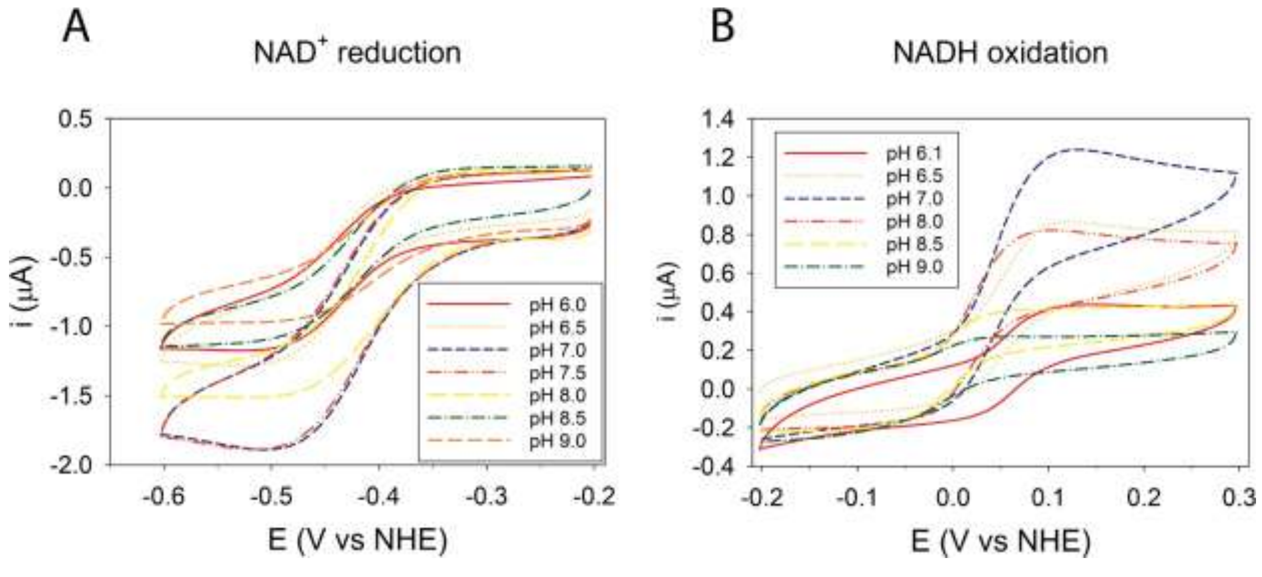

Figure S8. pH-dependent CVs of (A) methyl viologen (60 µM), NAD^+ (5 mM) and FdsBG (034 µM) and (B) methylene blue (20 µM), NADH (500 µM) and FdsBG (0.34 µM) and as a function of pH. See experimental section for buffer compositions (each 50 mM). Scan rate 5 mV s^{-1}.

Table S1. All Electrochemical simulation parameters

**Diffusion coefficients**	***Electrochemical parameters***	
$MV^{2+}/MV^{+\cdot}$ 3.8×10^{-6} cm^2 s^{-1}	$k_{0,MV}$ 1.0×10^{-2} cm s^{-1} (n = 1, α = 0.5)	
MB^{+}/MBH 2.1×10^{-6} cm^2 s^{-1}	$k_{0,MB}$ 1.0×10^{-2} cm s^{-1} (n = 2, α = 0.5)	
$NAD^{+}/NADH$ 4.0×10^{-6} cm^2 s^{-1}	Double layer capacitance 2.0×10^{-5} F	
FdsBG(all forms) 5.9×10^{-7} cm^2 s^{-1}	Electrode surface area (A) 0.056 cm^2	
Mediator dependent rate constants and potentials		
$MV^{2+} + e^{-} \rightleftharpoons MV^{+\cdot}$	E' (pH 7.5) −0.43 V vs NHE	
$MB^{+} + 2e^{-} \rightleftharpoons MBH$	E' (pH 7.5) +0.06 V vs NHE	
$MV^{+\cdot} + FdsBG_{ox} \rightleftharpoons MV^{2+} + FdsBG_{int}$	k_3 3.5×10^{7} M^{-1} s^{-1}	k_4 *3.5 × 10*^{4} M^{-1} s^{-1}
$MV^{+\cdot} + FdsBG_{int} \rightleftharpoons MV^{2+} + FdsBG_{red}$	$k_3{}^{a}$ 3.5×10^{7} M^{-1} s^{-1}	$k_4{}^{b}$ *3.5 × 10*^{4} M^{-1} s^{-1}
$MB^{+} + FdsBG_{red} \rightleftharpoons MBH + FdsBG_{ox}$	k_5 1.6×10^{7} M^{-1} s^{-1}	k_6 *1.C × 10*^{3} M^{-1} s^{-1}
Mediator independent rate constants		
$FdsBG_{red} + NAD^{+} \rightleftharpoons FdsBG_{ox} + NADH$	k_{red} 2.0×10^{6} M^{-1} s^{-1}	k_{ox} 2.0×10^{6} M^{-1} s^{-1}

[a] k_3 assumed to be equal for reduction of $FdsBG_{ox}$ and $FdsBG_{int}$; [b] k_4 assumed to equal for oxidation of $FdsBG_{int}$ and $FdsBG_{red}$.
